# Tip Cells Act as Dynamic Cellular Anchors in the Morphogenesis of Looped Renal Tubules in *Drosophila*

**DOI:** 10.1016/j.devcel.2013.09.020

**Published:** 2013-11-11

**Authors:** Helen Weavers, Helen Skaer

**Affiliations:** 1 Department of Zoology, University of Cambridge, Downing Street, Cambridge CB2 3EJ, UK

## Abstract

Tissue morphogenesis involves both the sculpting of tissue shape and the positioning of tissues relative to one another in the body. Using the renal tubules of *Drosophila*, we show that a specific distal tubule cell regulates both tissue architecture and position in the body cavity. Focusing on the anterior tubules, we demonstrate that tip cells make transient contacts with alary muscles at abdominal segment boundaries, moving progressively forward as convergent extension movements lengthen the tubule. Tip cell anchorage antagonizes forward-directed, TGF-β-guided tubule elongation, thereby ensuring the looped morphology characteristic of renal tubules from worms to humans. Distinctive tip cell exploratory behavior, adhesion, and basement membrane clearing underlie target recognition and dynamic interactions. Defects in these features obliterate tip cell anchorage, producing misshapen and misplaced tubules with impaired physiological function.

## Highlights

• Tip cells stabilize elongating renal tubules by binding to specific muscle targets
• Tip cell anchorage antagonizes migration, producing excretory tubule looping
• Tip cell exploration and adhesion dynamics underpin regulated tubule morphogenesis
• Lack of continued anchorage results in deformed tubules with impaired function

## Graphical Abstract

**Figure:**
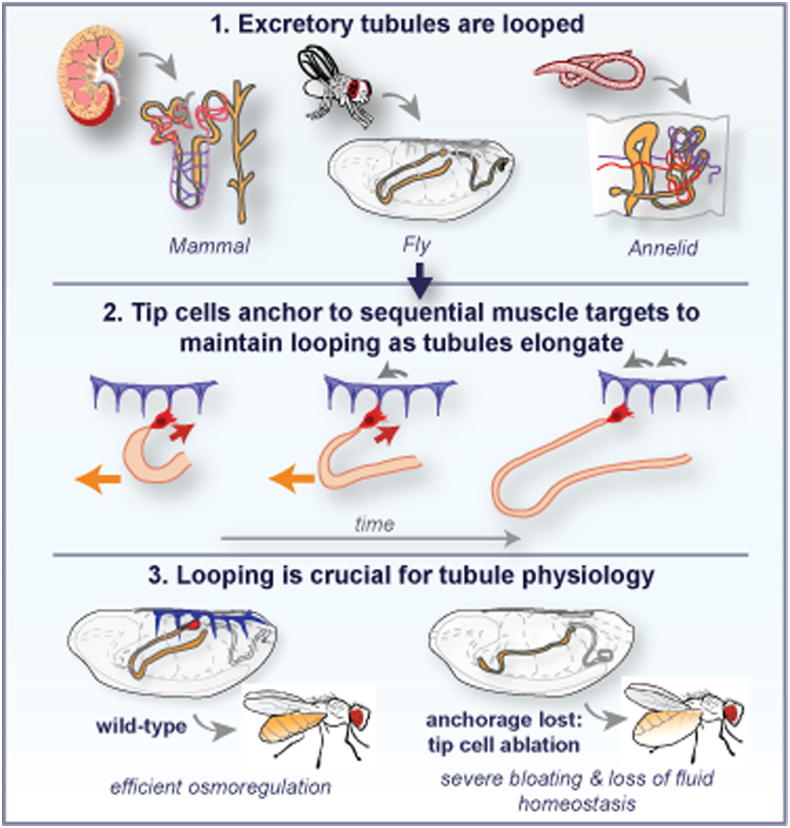

## Teaser

Renal tubules in *Drosophila* are looped structures that carry out excretion functions. Weavers and Skaer show that the tubules acquire their shape via specialized tubule tip cells that bind muscle targets to ensure looping. Binding is continuously required for initiation and maintenance of tubule morphology and function.

## Introduction

Many organs are built around branched networks of tubular epithelia, precisely organized in three dimensions. Seminal studies have uncovered the processes underlying the spatial regulation of tubule branching (Sternlicht et al., 2006; Affolter et al., 2009; Costantini, 2010). Branching is frequently followed by tubule extension, which is also highly regulated in space. In many systems, tubule branching and/or extension is regulated by the activity of specialized tip cells at the distal ends of tubules. During vertebrate angiogenesis, tip cells guide newly sprouted vessels toward angiogenic stimuli (Gerhardt et al., 2003) and, in the generation of both mammalian and fly respiratory systems, cells at the branch tips receive high levels of FGF and lead outgrowth toward the source of growth factor (Ribeiro et al., 2002; Caussinus et al., 2008; Bellusci et al., 1997). The morphogenesis of *Drosophila* salivary glands and the nematode gonad also depend on the activity of distally placed tip cells (Bradley et al., 2003; Blelloch et al., 1999). Indeed, tip cells act as organizers in primitive multicellular systems; in *Dictyostelium*, cells at the tip of the “slug” act to guide its migration and to control the differentiation of its constituent cells (Rubin and Robertson, 1975).

Whereas tubule elongation in the majority of systems produces an extensive, branched network, in other organs, such as the kidney (or regions of the vascular system), tubules develop a looped morphology. In this architecture, the proximal and distal regions (or for blood vessels, branch points) remain relatively close to one another, and elongation produces an extended U shape, characteristic, for example, of the loop of Henle. This morphology is also seen in the stereotypically positioned renal (Malpighian) tubules of *Drosophila*. The invariant architecture and positioning of insect renal tubules were observed a long time ago; Wigglesworth (1939) noted that “in spite of their great length and twisted course their arrangement is extraordinarily constant in a given species.” As insects have an open circulatory system, primary urine cannot be formed by pressure filtration, so the precise arrangement of renal tubules in the body cavity is important for comprehensive clearance of hemolymph-borne toxins and for effective ionic and osmotic homeostasis.

Insect tubules have distally placed tip cells that regulate tubule cell proliferation but persist long after cell proliferation is complete (Skaer, 1989, 1992a, 1992b). We have therefore used the renal system of *Drosophila* to investigate the role of tip cells in the spatially regulated outgrowth of tubules.

**Figure 1:**
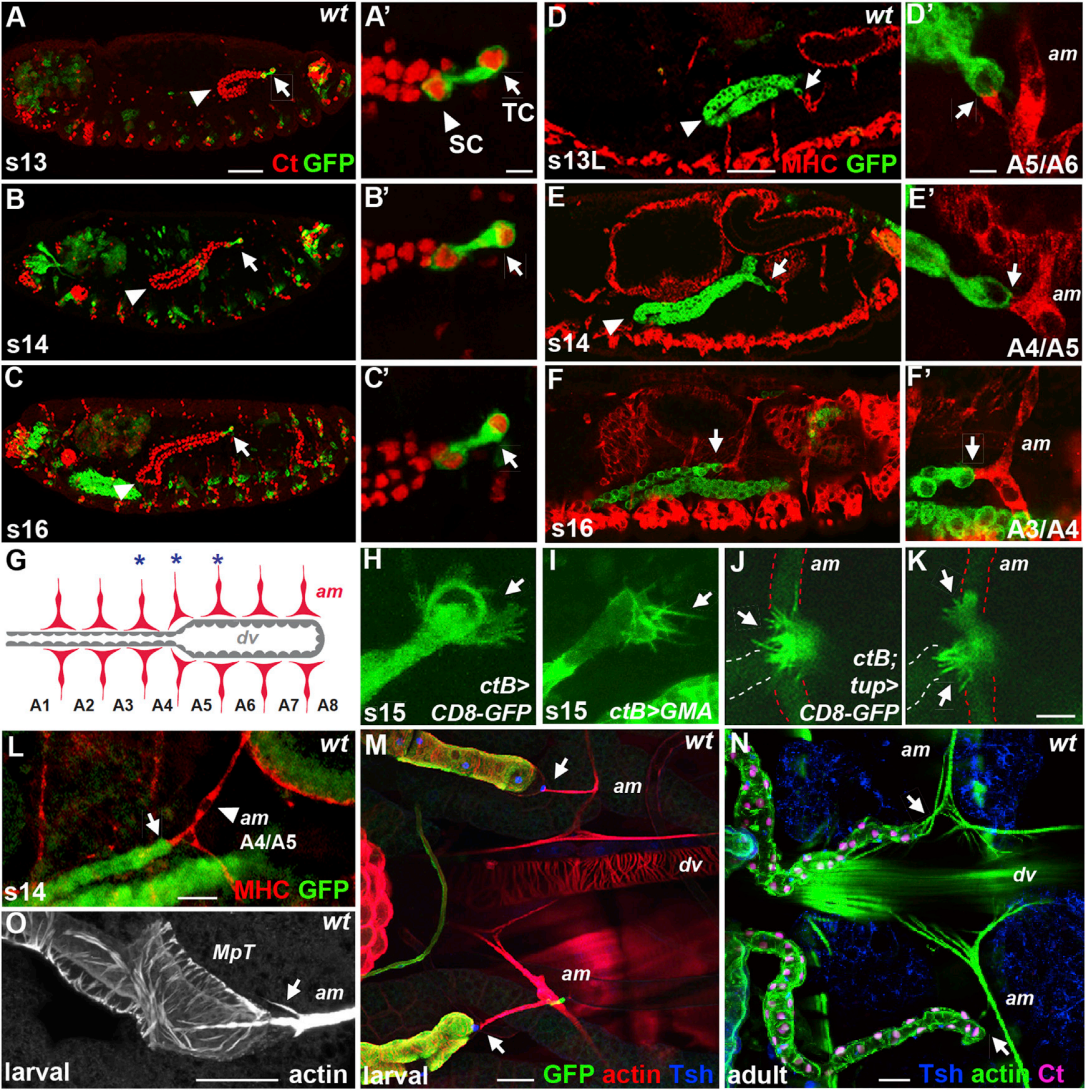
Tip Cells Contact Alary Muscle Targets (A–C) Wild-type stage 13–16 embryonic anterior tubules (Ct, red) navigate along precise routes, led by the kink region (arrowheads). Tip cells (green; *aseGal4>mCD8GFP*) protrude from distal tubule ends (arrows) but sibling cells do not (A′, arrowhead). (D–F) Tip cells (tubules, green; *ctBGal4>mCD8GFP*) contact successive alary muscle pairs (MHC, red) at the A5/A6, A4/A5, and A3/A4 segment boundaries (arrows). (G) Seven pairs of embryonic alary muscles (red) from A1/A2 to A7/A8 attach the dorsal vessel (gray) to lateral epidermis. Asterisks (blue) indicate tip cell contacts. (H–K) Protrusive tip cell membrane activity (H–I) associated with alary muscle interaction (J and K, arrows). Tubules and muscles are labeled with membrane CD8-GFP (H, J, and K) or moesin-GFP for actin (I). (L) Alary muscles (MHC, red) deformed (arrowhead) at the point of tip cell contact (arrow, *ctB-eGFP*). (M–O) Tip cell-muscle attachments (arrows) in third-instar larvae (M; *ctBGal4>mCD8GFP*; O) and adults (N). Actin-rich alary muscle fibers extend over the tip cell surface (O, arrow). Phalloidin labels actin (red, M; green, N; white, O); Tsh (blue, M and N) and Ct (magenta, N) label tip and tubule cell nuclei, respectively. TC, tip cell; SC, sibling cell; am, alary muscle; dv, dorsal vessel. Scale bars represent 50 μm (A–F), 5 μm (A′–F′ and H–K), 10 μm (L), and 30 μm (M–O). For all figures, anterior is left and dorsal is top in lateral perspectives.

*Drosophila* Malpighian tubules arise from the embryonic hindgut, budding out as four short tubular structures. Each bud enlarges by cell division, regulated by cells of the tip cell lineage, which secrete the EGF ligand Spitz to promote regionally restricted cell division (Kerber et al., 1998; Sudarsan et al., 2002). It is only after the completion of cell proliferation that the tubules elongate. Strikingly, as they lengthen, their extension through the body cavity follows a highly stereotypical path, with two projecting into the anterior body cavity and two into the posterior. We have shown that this precision results in part from guided morphogenesis through the reception of cues secreted from tissues adjacent to their navigation route (Bunt et al., 2010). Although these cues act to guide a specific region of the tubules (the “kink” region of the loop where the anterior tubules bend back on themselves; see Figure 1), the entire tubule is stereotypically positioned, suggesting that other regions regulate tubule architecture and positioning.

In this paper, we analyze the role of the distal tips of the anterior tubules in the morphogenetic movements that determine their looped shape and final positions in the body cavity. We show that tip cells make specific contacts with target tissues as the tubules elongate, and maintain their final targets into adult life. We demonstrate that the formation of both transient and final contacts is crucial for the normal looped architecture of the tubules. We present a hypothesis to explain the interactions that normally regulate tubule shape and account for the misrouting phenotypes we find when either tip cells or their targets are lost. Through genetic analysis and live imaging, we show that the tip cell’s lack of basement membrane and its active protrusive membrane activity and expression of specific adhesion molecules are characteristics that underlie its ability to interact with its targets, thereby ensuring the reproducibility of tubule morphology. As the mature shape of fly renal tubules is reminiscent of excretory tubules from annelid nephridia to mammalian nephrons, the regulatory mechanisms we describe could be widely relevant in nephrogenesis.

## Results

As the Malpighian tubules elongate, during stages 13–16 of embryogenesis, they course through the body cavity, taking up characteristic and markedly invariant positions by the end of embryogenesis (Figures 1A–1C; Bunt et al., 2010). Tip cells, at the distal end of each tubule, persist through tubule elongation (Figures 1A′–1C′), and by the end of this process contact specific tissues; posterior tip cells contact paired nerves that run up either side of the hindgut visceral muscle (Hoch et al., 1994), and anterior tip cells contact the paired alary muscles at the A3/A4 segmental boundary—one of the seven pairs of segmentally reiterated contractile alary muscles, which support the heart, linking it to the lateral body wall (Figures 1F and 1G). Our analysis focuses on the morphogenesis of the anterior tubules.

As the anterior tubules elongate, they form a tightly looped structure, with the point of maximum curvature, or kink, leading forward movement (Figures 1A–1C; Bunt et al., 2010). The distal tip of each tubule lies more posteriorly but moves forward as tubule elongation progresses (Figures 1A–1C). The tip cells initially contact the paired alary muscles at the A5/A6 segmental boundary, later contacting the muscles at A4/A5 before binding to their final targets at A3/A4 (Figures 1D–1F and 1D′–1F′). Establishing these contacts occurs in a 150 min window and is associated with dynamic behavior of the tip cells; the surface membranes show highly protrusive activity through the formation of actin-rich filopodia and lamellipodia (Figures 1H and 1I; Movie S1 available online). This dynamic filopodial activity is associated with exploration of each alary muscle as a contact is made (Figures 1J and 1K; Movies S2 and S3). Live imaging indicates that tip cells remain attached to their transient contacts for approximately 30 min before exploring the adjacent, more anterior alary muscle, detaching and rebinding to the new target, a process that takes about 25 min. Even the early temporary contacts are able to transmit force, as shown by the deformation of the alary muscle that occurs before each contact is finally broken and the tubule tip cell recoils forward to establish contact with the next alary muscle (Figure 1L; Movie S2).

Once the tip cells reach their final alary muscle targets a permanent contact is made, so that the tip cells remain attached to the A3/A4 alary muscles throughout larval (Figure 1M) and adult life (Figure 1N). The alary muscles develop long, actin-rich side extensions (Figure 1M) along the tip cell surface (Figure 1O).

### Manipulation of Tip Cell Number Perturbs Morphogenesis

**Figure 2:**
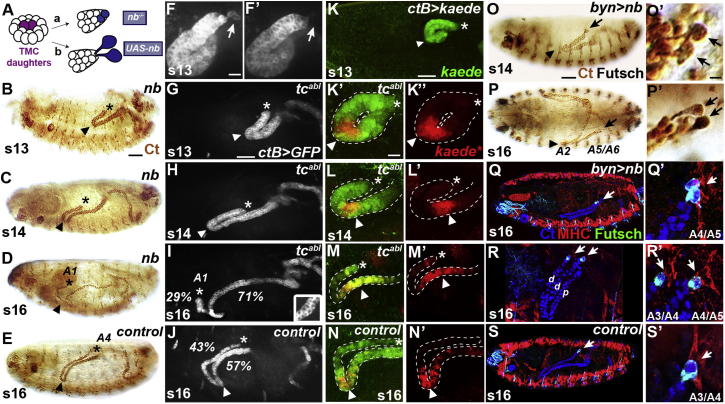
Tip Cells Are Required to Establish Anterior Tubule Architecture (A) Removal or overexpression of the Notch inhibitor *numb* (*nb*) gives tubules that lack (a) or possess two tip cells (b), respectively. (B–E) Anterior tubule architecture (Ct, brown) in *nb* mutants lacking tip cells (B–D; cf. control, E). Distal tubule ends move further anteroventral (B–D, asterisks) and the kink shifts distally (B–D, arrowheads). (F–J) Identical tubule defects (G–I) following laser ablation of anterior tip cells (arrows) at stage 13 (F, before; F′, after ablation; *ctBGal4>mCD8GFP*). Distal regions of control tubules contain 43% of cells (J); cf. 29% after tip cell loss (I). Anterior tubules fully elongate with two cells surrounding the lumen by stage 16 (I, inset). (K–N) Kink region (arrowheads) labeled by local activation of the photoconvertible fluorophore Kaede (kaede^∗^, red; K–K′′, arrowheads) at stage 13. Cells rapidly move out of the kink (L–M′, arrowheads) after tip cell ablation (asterisk), but labeled cells of control tubules do not (N and N′, arrowheads). (O–S) *bynGal4>nb* tubules with two tip cells (Futsch, black, O and P; green, Q–S; tubule cells Ct, blue) stall posteriorly with both tip cells attached to posterior alary muscles (MHC, red; Q′, arrow). Tip cells in branched tubules (R) attach to separate alary muscles (R′, arrows). Tip cells of control tubules (S) attach to the A3/A4 muscle (S′). TMC, tip mother cell; abl, tip cell ablation; d, distal; p, proximal. Scale bars represent 50 μm (B–E, G–K, and O–S), 10 μm (F and K′–N′), and 5 μm (O′–S′). See also Figure S1.

Previous work has shown that tip cells arise by single division of a progenitor cell, which is itself selected through lateral inhibition from a small group of competent cells in each tubule. Both progenitor cell daughters have equivalent tip cell potential but only one adopts the primary tip cell fate, its sibling being repressed through Delta/Notch signaling. Reception of this inhibitory signal is biased by the asymmetric inheritance of the Notch inhibitor Numb in the tip cell (Hoch et al., 1994; Wan et al., 2000; Sudarsan et al., 2002). If the activity of Notch in the tip cell lineage is increased, either by removing the function of *numb* (see Figure 2A) or by driving the activated Notch^intra^ receptor fragment in both daughter cells (Figure S1A), two sibling cells develop in the absence of tip cells. Conversely, if *numb* is hyperactive, two tip cells differentiate at the expense of the sister (sibling cell) fate (Figure 2A). In either situation, tubule cell division and tissue elongation occur (Wan et al., 2000; Ainsworth et al., 2000), as both cell types secrete the EGF ligand Spitz (Sudarsan et al., 2002). However, our observations now reveal that the final shapes and positions of the tubules in the body cavity are abnormal (Figures 2B–2D and 2O–2R; cf. Figures 2E and 2S).

In the absence of tip cells, the anterior tubule tips fail to contact any of the alary muscles, even though these muscles develop normally (Figure S1B), and the tubules become strikingly misshapen and mispositioned (Figures 2B–2D; Figure S1A). The elongating tubules move more rapidly forward so that by the end of embryogenesis, the distal tubule ends are located both more anterior (in A1) and further ventral in the body cavity. As a result, the normally tight kink region loosens and its position along the proximodistal axis of the tubule becomes progressively more distal (Figures 2B–2D; Figure S1A), so that by stage 16 the characteristic, tightly looped architecture of the tubule is lost (cf. Figure 2D with Figure 2E).

Cells in the kink region of the anterior tubules normally respond to Dpp guidance cues secreted by the dorsal epidermis, midgut, and gastric caeca, a process known to ensure normal tubule morphogenesis (Bunt et al., 2010). We asked whether these signals are active in *numb* mutants and found that DadGFP, a pathway target (Ninov et al., 2010; Weiss et al., 2010), was expressed in kink cells as in wild-type (Figures S1C and S1D). Further, tubule cells differentiate at the end of embryogenesis, transporting the nitrogenous waste product urate, which forms uric acid crystals in the tubule lumen (Figure S1E), showing that other aspects of tubule maturation are unperturbed.

We sought to confirm the loss-of-tip cell phenotype by laser ablating the tip cells of anterior tubules at stage 13 after cell division is complete (Figure 2F; Movie S4). The tubules elongate but are mispositioned exactly as in *numb* mutant embryos, with the distal regions lying more anteroventral with loose kink regions and no tip cell-alary muscle contacts (Figures 2G–2I; cf. Figure 2J; Movie S5). A similar phenotype is found if tip cell ablation is delayed until stage 14, after the first alary muscle contact has been made (Figure S1F), showing that each contact is important for tubule shape and positioning.

In the absence of tip cells, the tubules lose their looped structure as the kink region loosens and shifts distally so that the region of the tubule distal to the kink becomes shorter and the proximal region becomes concomitantly longer. We analyzed the number of cells in each region and measured their relative lengths in stage 16 embryos (Table S1). Compared to wild-type tubules (43% distal, 57% proximal; cell number), those lacking tip cells have 29% distal, 71% proximal (Figures 2I and 2J). These measurements suggest that in tubules without tip cells the kink becomes less stable. We tested this idea by expressing the photoconvertible fluorescent protein Kaede (Ando et al., 2002) throughout the tubules (Figure 2K) and UV irradiated just the kink region of stage 13 tubules to cause stable green-red photoconversion of Kaede (Figure 2K′). The movement of photoconverted cells confirms the relative stability of the kink region in control tubules with tip cells (Bunt et al., 2010; Figure 2N; Figures S1G–S1I). In contrast, if the tip cell is ablated immediately after photoconversion, cells of the kink region move into the proximal region more rapidly (Figures 2K–2M), resulting in shrinkage of the distal region and corresponding lengthening of the proximal region.

To test the effects of increasing the number of tip cells in each tubule, we overexpressed *numb* using an early hindgut/tubule driver, *byn*Gal4. Two tip cells differentiate in each tubule without any apparent alteration in the fates of other tubule cells (Ainsworth et al., 2000; Figure 2O′). Tubule cell division and tissue elongation occur normally, and the early outgrowth of the tubules during stage 14 is similar to wild-type (Figure 2O; cf. Figure 1B). However, the tubules fail to complete their forward navigation and stall in the posterior of the embryo, with the kink region in A2 (cf. T3 in wild-type), and the distal tips remain bound to their early alary muscle contacts in A5/A6 (45%, n = 20) or A4/A5 (40%, n = 20) (Figures 2P and 2Q; cf. Figure 2S). In rare cases, tubules with two tip cells branch, producing a duplicate distal region each with a single tip cell (Figure 2R), which form separate alary muscle contacts at the segmental boundaries A3/A4 and A4/A5 (Figure 2R′).

Together, these results show that normal tubule architecture depends on the allocation of a single tip cell in each tubule. The importance of tubule shape and positioning is indicated by the appearance of the flies that survive to adulthood after (genetic) tip cell ablation, in which two sibling cells are allocated in each tubule at the expense of tips cells (*acGal4>N*^*intra*^; 50%, n = 50). Adults become bloated with retained fluid by 3 days after eclosion and die prematurely, most likely due to failure of fluid homeostasis (Denholm et al., 2013; Figures S1J and S1K).

### Tubule Positioning Depends on Tip Cell Targets

**Figure 3:**
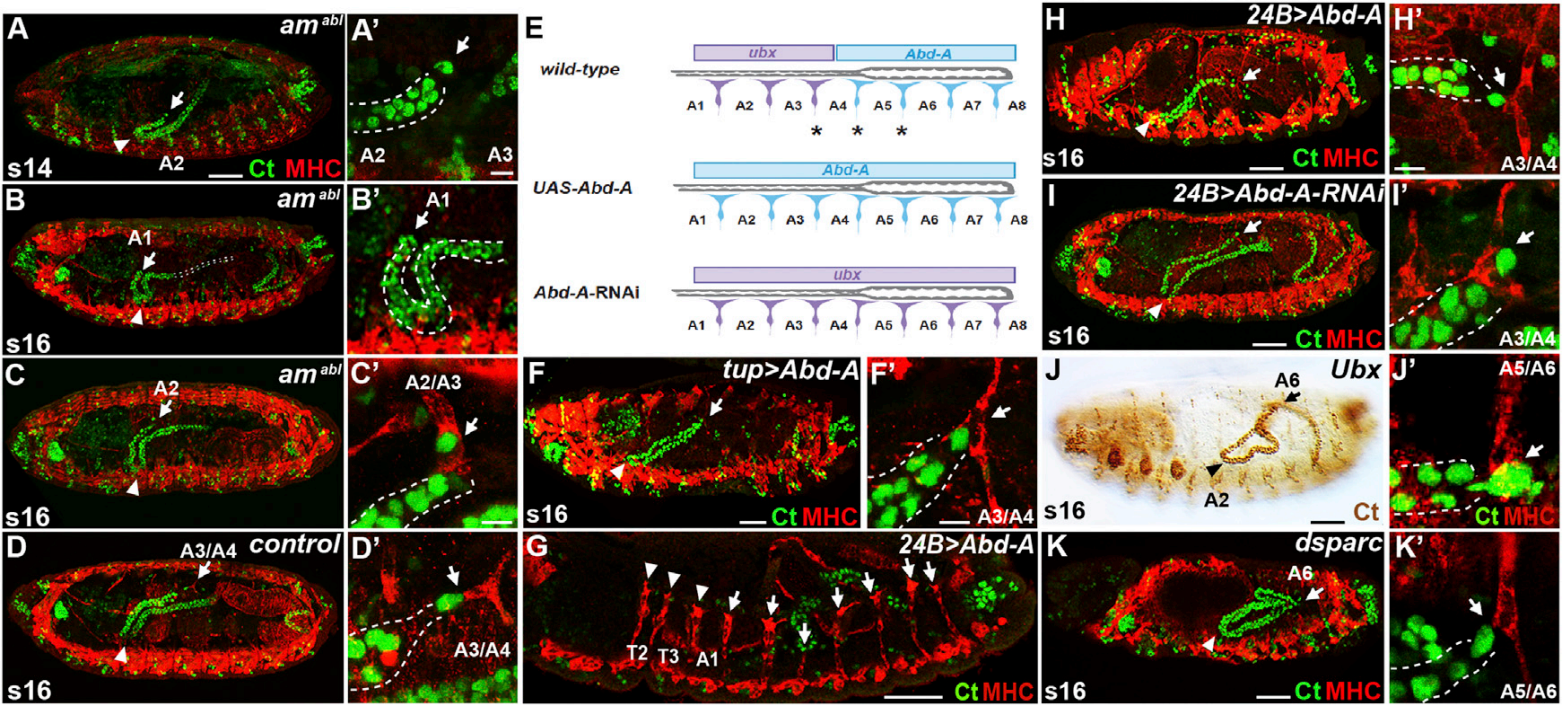
Alary Muscles Act as Distal Anchors during Tubule Morphogenesis (A–D) Ablation of the A3/A4, A4/A5, and A5/A6 alary muscles (MHC, red) perturbs anterior tubule architecture (A–C; control, D; Ct, green). Distal regions move further anteroventrally (A–C, arrows) and the kink moves distally (arrowheads); tip cells either fail to contact alary muscle targets (A′ and B′, arrow) or attach further anteriorly (C′, arrow). (E–I) Manipulation of alary muscle anteroposterior identity does not affect tip cell choice. Three anterior-most alary muscle pairs (A1/A2–A3/A4) express *Ubx* (purple) but not *Abd-A*. Four posterior-most alary muscle pairs (A4/A5–A7/A8) express *Abd-A* (blue) but not *Ubx*. Asterisks indicate alary muscle targets. Ectopic *Abd-A* expression (*tupGal4* in F; *24BGal4* in G and H) posteriorizes all alary muscles (E). *24BGal4>Abd-A* embryos possess three additional alary muscle pairs, T1/T2–T3/A1 (G, arrowheads). *24BGal4>*Abd-A^RNAi^ anteriorizes alary muscles (E). In all cases, tip cells (Ct, green) contact A3/A4 alary muscles (MHC, red) as normal (F′, H′, and I′, arrows). (J–K) In *Ubx* (J) and *dsparc* (K) mutants, anterior tubules (Ct, brown, J; green, J′ and K) stall with their kinks in A2 (arrowheads) and tip cells bound to posterior alary muscles (MHC, red) at A5/6 (J′ and K′, arrows). Scale bars represent 50 μm (A–D and F–K), 10 μm (A′ and B′), and 5 μm (C′, D′, F′, H′, and K′). See also Figure S2.

We asked whether tip cells influence tubule positioning through contact with their normal targets by laser ablating alary muscles in an otherwise intact embryo. We ablated all three alary muscles normally contacted by the anterior tubule tip cell on one side of the embryo (A5/A6, A4/A5, and A3/A4; Movie S6) and found that although all the tubules extend, only the tubule on the ablated side is misshapen and abnormally positioned (Figures 3A–3C; cf. internal nonablated control, Figure 3D). In most cases, the tubule position resembles the loss of the tip cell, with the kink loosening and lying further forward, the distal tip more anteroventral, and the tip cell failing to contact any target at all (Figures 3B and 3B′; 70%, n = 10). In other cases, the tubule extends too far anterior but the distal region remains dorsal and the tip cell contacts a more anterior alary muscle at A2/A3 (Figures 3C and 3C′; 30%, n = 10).

As the tip cell normally makes stereotypical contacts with specific alary muscles, we tested whether altering the segmental identity of alary muscles would affect target selection and tubule positioning. In wild-type embryos, the A4/A5 alary muscle and those posterior to it express *AbdA*, whereas A1/A2 to A3/A4 alary muscles express *Ubx* (Figure 3E; Figures S2A and S2B; LaBeau et al., 2009). We altered the expression of these homeotic genes, “posteriorizing” all alary muscle pairs by driving *AbdA* using the pan-alary muscle driver *tailup*Gal4, which also leads to the repression of *Ubx* in the anterior alary muscles (Figure 3E; Figures S2C and S2D). The anterior tubules develop normally, with the tip cells making their normal transitory and final contacts (Figures 3F and 3F′). We used the pan-mesodermal, early driver 24BGal4 to see whether a stronger driver might produce a phenotype. Even though *24BGal4>AbdA* results in the development of three pairs of ectopic alary muscles in T1/T2 to T3/A1 (Figure 3G), tip cells make their normal A3/A4 alary muscle contacts (Figures 3H and 3H′). We then “anteriorized” all alary muscles using 24BGal4 to express *AbdA-*RNAi, which abrogates its expression in posterior alary muscles and induces them to express *Ubx* (Figure 3E; Figure S2E). Again, the tubules develop normally, with tip cells making their normal contacts (Figure 3I).

These results show that tip cells do not sense the segmental identity of their alary muscle targets; they appear to make indiscriminate contact with alary muscles at each segmental boundary as they come into contact with them. Indeed, under certain conditions, they are able to make stable contacts with alary muscles in more anterior segments than normally occurs in wild-type (Figure 3C). This suggests that factors other than segmental identity dictate their final alary muscle target.

In normal development, the forward projection of the anterior tubules relies on interactions of the kink region with Dpp guidance cues, and in their absence the tubules stall in the posterior. In *Ubx*^*9.22*^ mutant embryos, the Dpp guidance cue from the midgut visceral mesoderm is lost (Tremml and Bienz, 1989) and the tubules fail to navigate forward past the midgut (Bunt et al., 2010). We find that in these embryos, the tip cells remain stably attached to their initial posterior alary muscle target at A5/A6 (Figure 3J). However, as anterior alary muscles (A1/A2 to A3/A4) are variably missing in *Ubx*^*9.22*^ mutants (LaBeau et al., 2009), we also analyzed the phenotype of *dsparc* mutant embryos in which alary muscle development is unaffected but the reception of guidance cues is lost (Bunt et al., 2010) and, just as in *Ubx*^*9.22*^ mutant embryos, the tip cells make stable contacts with the A5/A6 alary muscles (Figures 3K and 3K′).

These results indicate that the final tip cell target is set by the balance of opposing interactions: the Dpp-dependent forward tubule movement, which pulls the tip cell from one alary muscle to the next, and tip cell-muscle contacts, which act to stabilize tubule position more posteriorly.

### Tip Cell Morphology Determines Tubule Positioning

**Figure 4:**
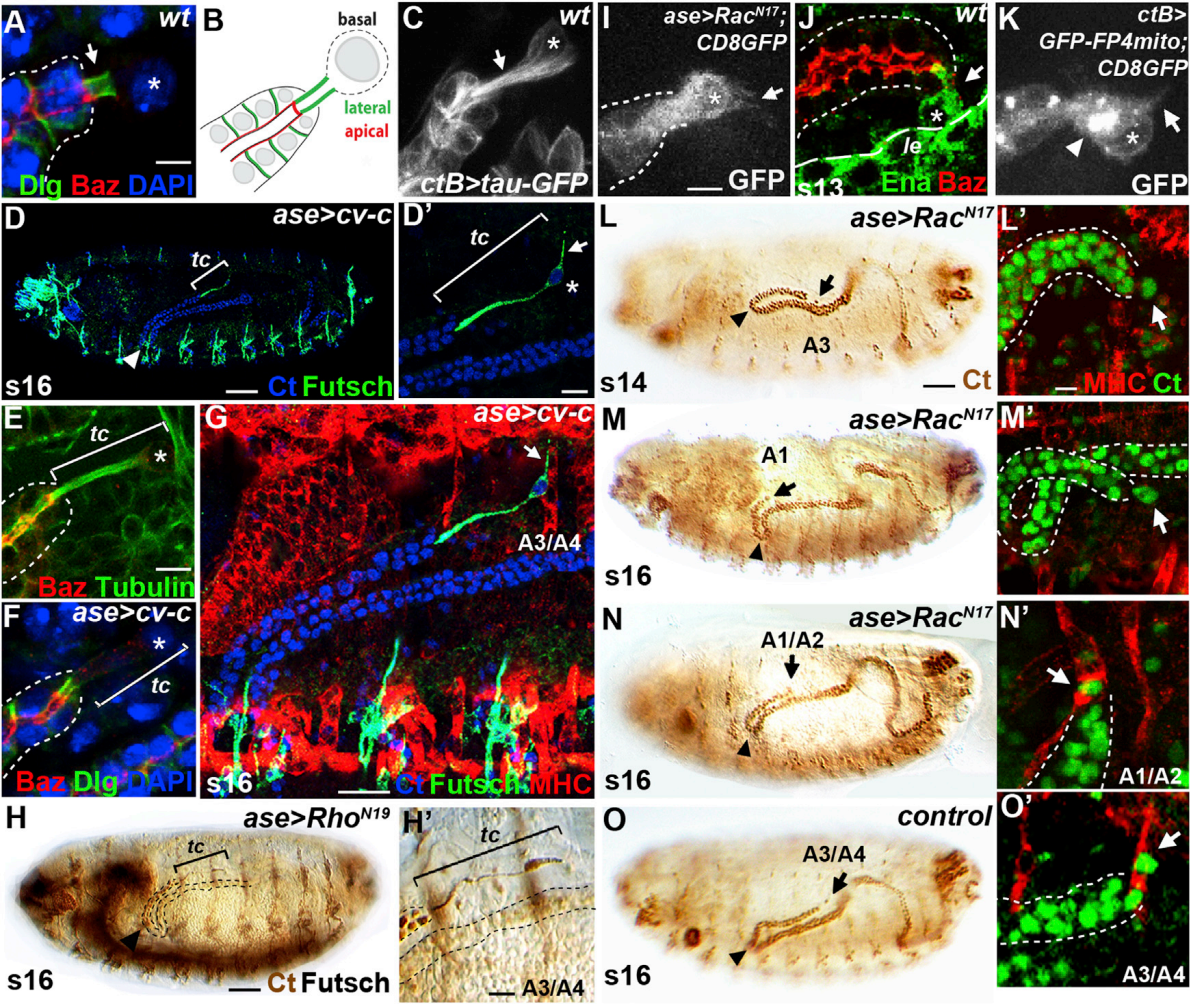
Tip Cell Morphology and Protrusive Activity Regulate Anterior Tubule Architecture (A–C) Tip cell apicobasal polarity. Baz (red) localizes apically at the luminal membrane (A and B). Dlg (green) localizes laterally at the stalk membrane (arrow, A and B). DAPI (blue) labels tip (asterisk) and tubule cell nuclei. Microtubules (C, white; *tau-GFP*) are concentrated in the stalk (arrow) and encapsulate the nucleus (asterisk). (D–H) Inactivation of tip cell RhoA by overexpressing RhoGAP *cv-c* (D–G) or dominant-negative *Rho*^*N19*^ (H) produces tip cells (tc) with abnormally long stalks (brackets), enriched in microtubules (E; Tubulin, green) and derived from basal membrane lacking Baz and Dlg (F). Elongated tip cells (Futsch, green), including membrane extensions basal to the tip cell nucleus (D′ and G, arrows), contact the alary muscle (MHC, red) at the A3/A4 boundary (G, arrow), and the tubules (Ct, blue, G; brown, H) move further anteroventral (G and H). (I–O) Tip cell membrane protrusions are significantly reduced by expression of dominant-negative *Rac*^*N17*^ (I) or Ena inactivation (K, arrow; *ctB>GFP-FP4mito*). Ena (green) is expressed in tip cells (asterisk) from stage 13 (J, arrow) at similar levels as epidermal leading-edge cells (*le*). GFP-FP4mito (DNEna) accumulates in cytoplasmic puncta (K, arrowhead). Without protrusive activity, tip cells (arrows, L–N) fail to attach to alary muscle targets (MHC, red, L′ and M′) or bind muscles further anterior (N′, arrow). Distal tubule ends (arrows) move further anteroventrally and the kink (arrowheads) moves distally (cf. L–N with O). Scale bars represent 5 μm (A–C, E, F, and I–K), 50 μm (D, H, and L–O), and 10 μm (D′, G, H′, L′–O′).

Tubule tip cells adopt a specialized morphology. They are apicobasally polarized with a small apical domain, separated from the extended basolateral domain by an adherens junction (Figures 4A and 4B). The lateral membrane encloses microtubule-enriched cytoplasm, forming the stalk (Figure 4C), so that the cell body projects from the tubule tip. We have previously shown that the length of this stalk region is influenced by the activity of a RhoGAP, Crossveinless-c (Cv-c). Overexpression of *cv-c* in the tip cell lineage results in a dramatic increase in stalk length (from 10 to >50 μm) (Denholm et al., 2005; Figure 4D). The elongated stalk is highly enriched with microtubules (Figure 4E) and is an extension only of the basal membrane, as the lateral membrane marker Discs large (Dlg) is absent from the extended stalk (Figure 4F).

We asked whether increasing the length of the tip cell stalk might affect the positioning of the tubules in the body cavity. The elongated tip cells of anterior tubules make contact with their normal final A3/A4 alary muscle targets, but the tubule kink regions extend further anteroventral than in wild-type (Figures 4D and 4G; see Table S1). To check that the increase in Cv-c acts through inhibition of its known target in tubule cells, the Rho GTPase RhoA, we expressed a dominant-negative form of RhoA in the tip cell lineage and found the same phenotypes, both in extension of the tip cell and misplacement of the anterior tubules (Figures 4H and 4H′).

These results confirm that there is a two-way interaction between tubule extension and tip cell position. Although in normal development the guided forward extension of the tubule delivers the tip cell to the A3/A4 alary muscle, the presence of a long tip cell stalk, rather than forcing the tip cell to make a more posterior alary muscle contact, allows the tubule to extend further into the anterior, with the tip cell making its normal A3/A4 alary muscle contact.

### Tip Cells Have Special Attributes

Live imaging reveals that tip cells actively explore their environment, forming filopodia and lamellipodia as they establish target contacts (Figures 1H–1K; Movie S2). These membrane extensions are formed by the dynamic modulation of the actin cytoskeleton and can be repressed by expressing a dominant-negative construct of the Rho GTPase Rac in tip cells (Figure 4I; average filopodial length: control, 7.2 μm ± 1.5 [SD], n = 7 tip cells; cf. Rac^N17^, 0.9 μm ± 0.5, n = 19 tip cells) (Nobes and Hall, 1995). We also found that the actin anti-capping factor Enabled is expressed in tip cells (Figure 4J) and that membrane protrusions were repressed when Enabled was inactivated by expressing the FP4mito construct that acts as a dominant negative (Gates et al., 2007) (Figure 4K; control, 7.2 μm ± 1.5; cf. 1.7 μm ± 0.8, n = 12 tip cells). It is striking that the lack of protrusive activity is correlated in the majority of cases with the failure of tip cell-alary muscle connections and the same tubule mispositioning as seen in the absence of tip cells or alary muscle targets (cf. Figures 4L and 4M with Figure 4O; see Table S1). In a few cases, tip cells do make muscle contact, but more anteriorly, with the A1/A2 alary muscle (Figure 4N).

### Tip Cells Lack a Basement Membrane

**Figure 5:**
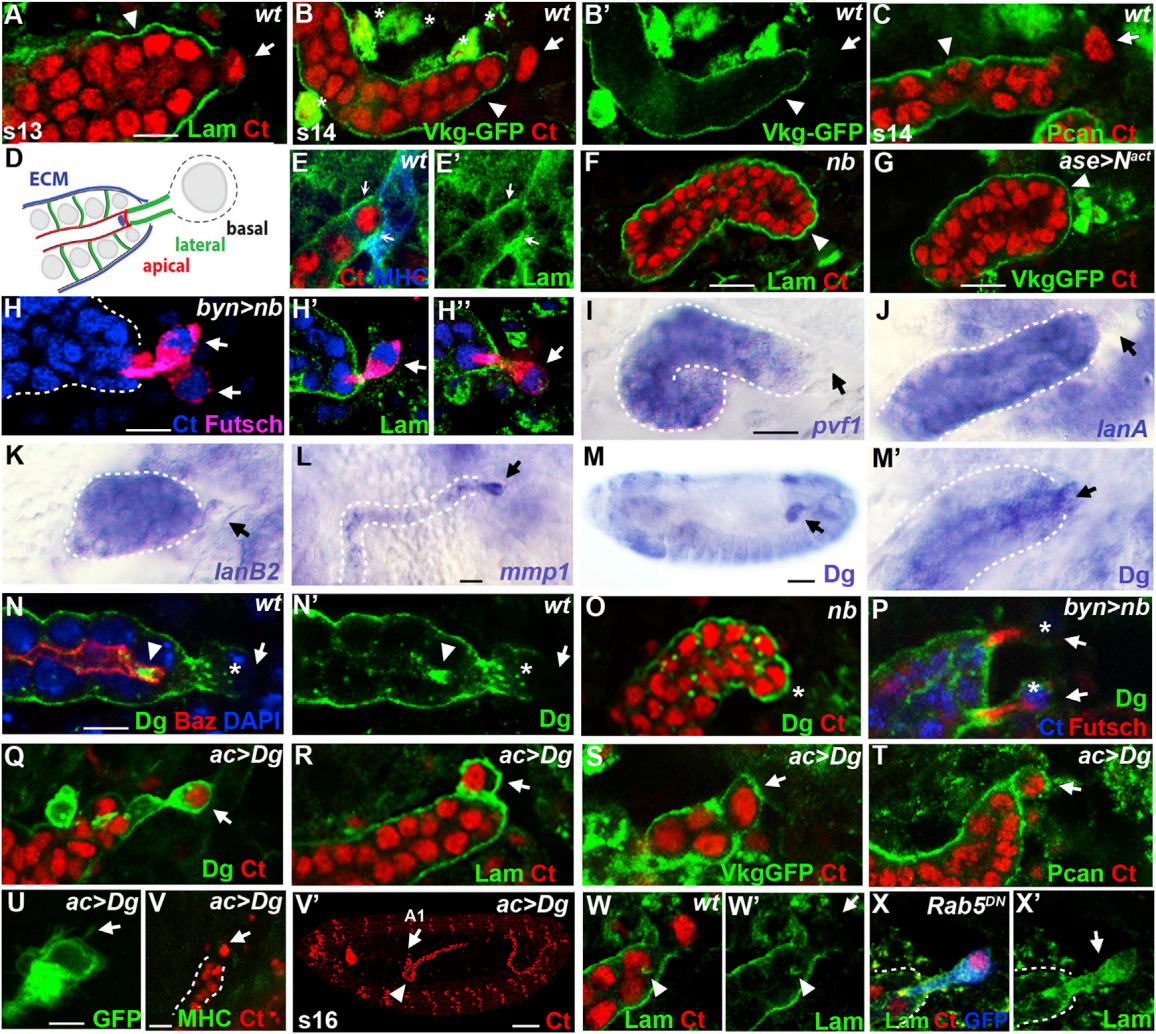
Tip Cells Lack a Basal Extracellular Matrix Sheath (A–E) Embryonic tubules (Ct, red) are ensheathed (arrowheads) in laminin (green, A), collagen IV (Viking, green, B and B′), and perlecan (green, C), but until attached to their final targets, tip cells lack this sheath (A–C, arrows; D, schematic). Laminin (green, E) localizes to the final tip cell-alary muscle junction (arrows, Ct, red; MHC, blue). (F and G) Distal ends of tubules lacking tip cells (arrowheads; F, *nb* mutant; G, *ase>N*^*act*^) encapsulated in a sheath of laminin (green, F) and collagen IV (Viking; green, G). (H) Ectopic tip cells (arrows; Futsch, magenta) in *byn>nb* tubules (Ct, blue) lack an extracellular matrix sheath (laminin, green; each tip cell shown in H′ and H′′). (I–L) Embryonic tubules express platelet-derived growth factor/vascular endothelial growth factor ligands *pvf1–3* (*pvf1* in I) and laminin subunits *lanA* (J) and *lanB2* (K) but tip cells do not (arrows, I–K, in situ hybridization). Tip cells express *mmp1* from stage 15 (L, arrow). (M–P) Dystroglycan in embryonic tubules, including the tip cell (in situ hybridization; tip cell, arrow, M and M′). Whereas Dg protein localizes basally in tubule cells (green, N and N′, arrows), in tip cells it localizes in a cap over the apical membrane (N and N′, arrowheads; tip cell basal membrane, arrow; nuclei [DAPI, blue], apical membrane [Baz, red]). Basal Dg (green, O and P) ensheathes the distal ends (asterisk) of *nb* mutant tubules (Ct, red) lacking tip cells (O) but is virtually absent from ectopic tip cells (asterisks) in *byn>nb* embryos (P, arrows). (Q–V) Tip cell-specific overexpression of *dg* causes basal accumulation (green, Q, arrow) of laminin (green, R), collagen IV (Viking; green, S), and perlecan (green, T) over the tip cell surface (R–T, arrows). Tip cell filopodia are reduced (U, arrow) and tip cell attachment to muscle targets (MHC, green) is disrupted (V, arrow); tubules (Ct, red) are mispositioned anteroventrally with their distal ends in A1 (V′, arrow) and the kink region further distal (V′, arrowhead). (W–X) Laminin (green) accumulates apically in wild-type tip cells (W and W′, arrowheads) but is absent basally (W′, arrow). Laminin (green, X and X′) accumulates in the cytoplasm (arrow) of tip cells expressing dominant-negative Rab5 (*ac>Rab5*^*DN*^). Scale bars represent 10 μm (A–L, M′–T, and V–X), 50 μm (M and V′), and 5 μm (U).

The developing tubules are ensheathed in a basement membrane (BM), which contains two collagen IVs (Viking, Cg25C), laminin, and perlecan (Bunt et al., 2010). Tubule cells secrete Pvf ligands from stage 11 to attract a subset of hemocytes, which secrete collagen IV, laminin, and perlecan around the tubules, although the tubule cells themselves also express laminin (Bunt et al., 2010). As the laying down of an extracellular matrix around the tip cells might impede their exploratory activity, we examined the presence of BM at the tubule tips. We found that tip cells remained free of collagen IV, laminin, and perlecan throughout tubule morphogenesis, only becoming invested by the BM after making their final target muscle contacts (Figures 5A–5D; final contact, Figure 5E). The absence of BM at the tubule tips is dependent on the presence of tip cells. In *numb* mutant embryos that lack tip cells or when *N*^*act*^ is driven in the tip cell lineage, the BM ensheathes the whole tubule (Figures 5F and 5G), and when *numb* is overexpressed, both tip cells remain free of extracellular matrix (Figure 5H).

We wondered how tip cells regulate their BM covering and found that they do not express the Pvf ligands and so are unlikely to attract hemocytes to their surface (Figure 5I; Pvf2 and Pvf3, data not shown). In contrast to neighboring tubule cells, tip cells do not express *lamininA* and *B* (Figures 5J and 5K). Thus, the secretion of BM components around tip cells is likely to be low. However, we also found that from stage 15, tip cells express the matrix degradation enzyme metalloproteinase MMP1 (Figure 5L), suggesting that any matrix deposited around the tip cells could be broken down.

In many tissues, the laminin receptor Dystroglycan (Dg) acts to stabilize the assembly of BM components (Henry and Campbell, 1998). We therefore analyzed Dg expression in tubules and found that whereas all tubule cells, including the tip cells, show clear mRNA expression from stage 13 (Figure 5M), the protein is found predominantly on the basal surface of all tubule cells except the tip cell (Figure 5N). The absence of basal Dg is a tip cell-specific characteristic, as in *numb* mutants that lack tip cells, Dg is found on the basal surface over the whole distal region of the tubule (Figure 5O), and when *numb* is overexpressed, producing two tip cells, neither has significant levels of basally localized Dg (Figure 5P).

We overexpressed Dg in the tip cell lineage using *ac*Gal4 and found that Dg is no longer excluded basally (Figure 5Q). This expanded distribution is associated with a complete covering of the tip cell by laminin, collagen IV (Viking), and perlecan (Figures 5R–5T). This ectopic BM interferes with the ability of the tip cell to develop filopodia (Figure 5U; average filopodial length: control, 7.2 μm ± 1.5 (SD), n = 7 tip cells; cf. Dg overexpression, 3.3 μm ± 0.9, n = 14 tip cells). As a result, tip cells do not make their alary muscle contacts (Figure 5V), and tubules fail to adopt their normal shape and position in the body cavity (Figure 5V′).

Careful observation shows that tip cells are not devoid of Dg; there is a concentration of protein on the small apical surface (Figures 5N and 5D). This apical distribution of Dg could result from transcytosis of any basally inserted protein. Indeed, we find similar apical concentrations of laminin (Figure 5W), suggesting that the tip cell might be actively clearing any BM deposited over its surface. This would involve the uptake and transcytosis of basally located Dg and associated BM components. Rab5, a small GTPase, promotes the fusion of endocytic vesicles to the early endosome, and has been shown to mediate transcytosis of BM components in border cells (Olkkonen and Stenmark, 1997; Medioni and Noselli, 2005). We drove the expression of a GFP-tagged dominant-negative DRab5 (Drab5^s43N^; Wucherpfennig et al., 2003) in the tip cell lineage. This construct impairs the fusion of endocytic vesicles to the endosome, resulting in the accumulation of endocytic vesicles in the cytoplasm (Bucci et al., 1992) and the failure of trafficking from the early endosome (Zerial and McBride, 2001). By stage 15, tip cells expressing Drab5^s43N^ contained high levels of laminin in the cytoplasm (Figure 5X), suggesting that laminin is normally taken up and further trafficked from tip cell endosomes, perhaps to the degradative pathway but also to the apical surface.

### Tip Cells Show Dynamic Adhesive Properties

**Figure 6:**
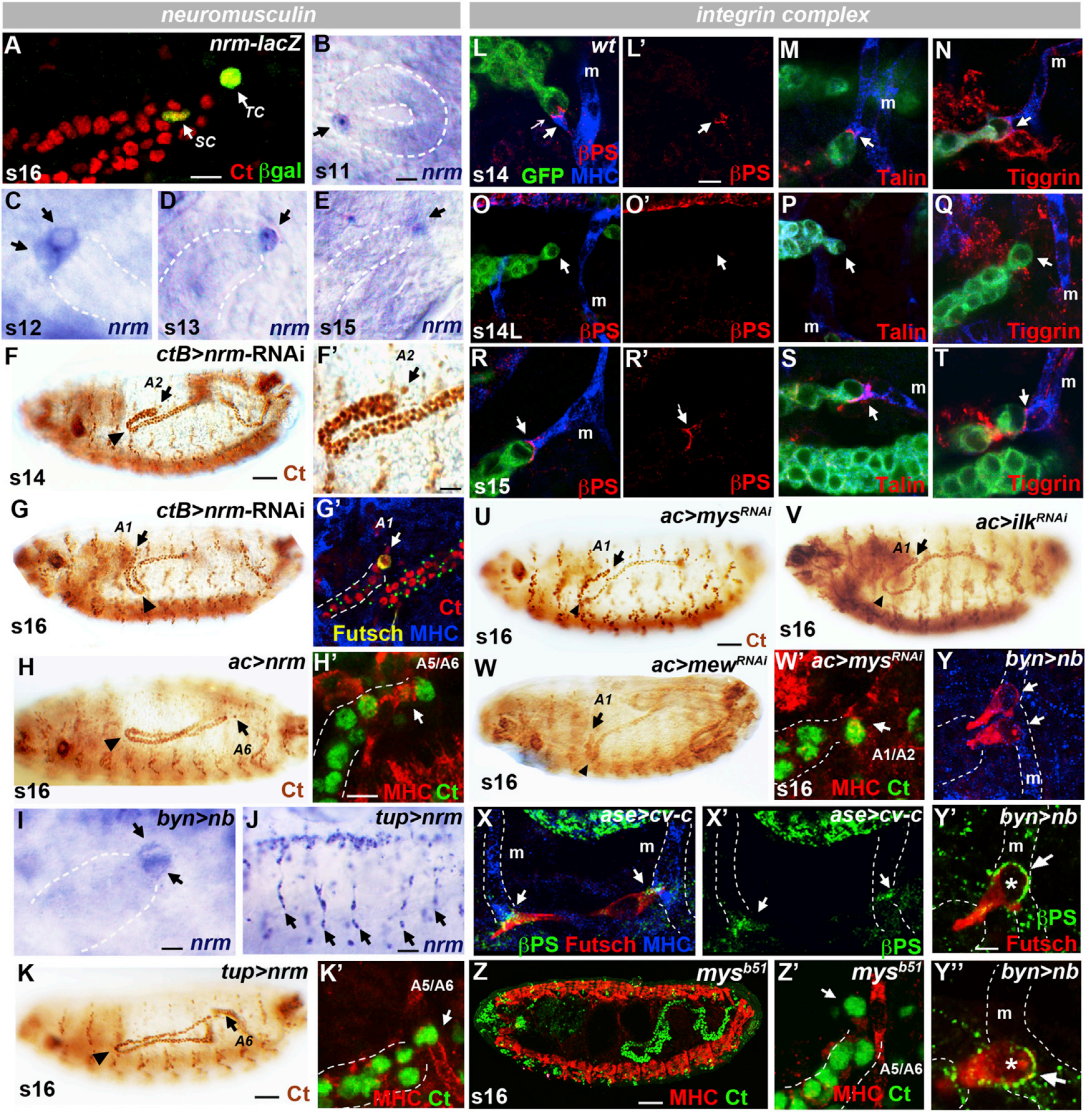
Tip Cell-Alary Muscle Interactions Require Neuromusculin and Integrin Adhesion (A–E) Neuromusculin is expressed in the tip cell lineage; *nrm-lacZ* reporter A37 (β-gal, green; A, arrows) and in situ hybridization (B–E). *nrm* is detected in the progenitor cell (B, arrows) and both daughters (C, arrows) before refining to the tip cell (D and E, arrows). TC, tip cell; SC, sibling cell. (F and G) In the absence of *nrm* (*ctB>nrm*^*RNAi*^), tubules (Ct, brown) move further anteroventrally (F and G, arrows), and tip cells (Futsch, green, G′) fail to attach alary muscle targets (MHC, blue; G′, arrow). Arrowheads, kink. (H) Overexpression of *nrm* in the tip cell lineage causes tubules (Ct, brown, H) to stall posteriorly with tip cells (arrows) bound to A5/A6 alary muscles (MHC, red, Ct, green, H′). (I) Ectopic tip cells in *byn>nb* tubules express *nrm* (arrows). (J and K) Ectopic expression of *nrm* in embryonic alary muscles (blue, J, arrows) causes tubules (Ct, brown, K, green, K′) to stall posteriorly with tip cells (arrows) bound to A5/A6 alary muscles (MHC, red, K′). (L–T) βPS integrin (red, L, O, and R), talin (red, M, P, and S), and tiggrin (red, N, Q, and T) localize to each tip cell-alary muscle junction (L–N and R–T, arrows) but are absent from the tip cell surface in the absence of muscle attachment (O–Q, arrows). (U–W) Tubules (Ct, brown) mislocalize anteroventrally following RNAi-mediated loss of tip cell βPS integrin *myospheroid* (*mys*; U and W′), *integrin-linked kinase* (*ilk*; V), or αPS1 integrin *multiple edematous wings* (*mew*; W). Arrowheads, kink. Tip cells (arrows) bind anteriorly to A1/A2 alary muscles (W′, Ct, green; MHC, red). (X and Y) βPS integrin (green; Futsch, red) localizes to points of tip cell-muscle contact (arrows) on long tip cells that bind multiple alary muscles (X and X′, arrows; MHC, blue) and ectopic tip cells of *byn>nb* tubules (Y–Y′′, arrows). Individual tip cells are shown in (Y′) and (Y′′). (Z) Tubules (Ct, green) stall posteriorly with tip cells (arrow, Z′) bound to posterior A5/A6 alary muscles (MHC, red) in embryos carrying an activating mutation in *mys* (*mys*^*b51*^). Scale bars represent 10 μm (A–E, F′–J, K′–T, and W′–X′), 50 μm (F–H, K, U–W, and Z), and 5 μm (Y′ and Y′′). See also Figure S3.

Tip cells clearly adhere to their final alary muscle targets, but our movies also show distortion of transient muscle targets as the tubules move forward, suggesting substantial adhesion between tip cells and the more posterior alary muscles (Movie S2). An enhancer trap line, A37 (Kania et al., 1993), in *neuromusculin* (*nrm*), which encodes a cell-adhesion protein, marks out the tip cell lineage (Hoch et al., 1994; Figure 6A). We sought to confirm *nrm* expression in tip cells by in situ hybridization and found expression in the tip cell progenitor and in its daughter cells, a pattern which resolves into the tip cell alone, persisting throughout tubule elongation (Figures 6B–6E). Driving *nrm*-RNAi in the tip cell lineage abrogates tip cell contact with alary muscles, resulting in mispositioning of the anterior tubules that resembles the loss of tip cells or alary muscles (Figures 6F and 6G; cf. with Figures 2D and 3A–3C). Nrm has been shown to act as a homophilic adhesion molecule in the fly nervous system (Kania et al., 1993; Kania and Bellen, 1995), but it is not expressed in tip cell target alary muscles, and driving *nrm*-*RNAi* in the alary muscles produces no mispositioning phenotype (data not shown).

In embryos in which *nb* is overexpressed and two tip cells develop, both express *nrm* (Figure 6I). This increase in tip cell adhesion might underlie the maintenance of more posterior tip cell-alary muscle contacts and the mispositioning of the tubules (Figures 2O and 2P). To test this idea, we increased the expression of *nrm* in tip cells (Figure 6H) and drove it ectopically in alary muscles (Figures 6J and 6K). In both experiments, distal tubule regions remained in the posterior with tip cells, stabilizing their first alary muscle contact in A5/A6 (Figures 6H′ and 6K′). Correspondingly, if *nrm* is driven in all tubule cells, other regions as well as the tip cells attach to alary muscles (Figures S3A and S3B). Thus, Nrm is both necessary and sufficient for target adhesion.

In other contexts where epithelial tissues make stable contacts with muscles, integrin-mediated adhesion plays a crucial role (Bökel and Brown, 2002; Levi et al., 2006; see Schejter and Baylies, 2010). We therefore analyzed the localization of integrins, one of their extracellular ligands, tiggrin, and a cytoplasmic partner, talin, at the tip cell-muscle junction. We find that integrin complexes are dynamically assembled when tip cells contact their target alary muscles (A5/A6 contact shown in Figures 6L–6N). Once released from a transient contact, integrins, tiggrin, and talin disappear from the tip cell surface (Figures 6O–6Q), reassembling when contact is made with the next target muscle. Once the final contact is made with the A3/A4 alary muscle, a stable accumulation of integrin, tiggrin, and talin forms (Figures 6R–6T). The association between the alary muscle and tip cell persists through larval and adult life (Figures 1M–1O; Figures S3C–S3E), with enrichment of laminin, collagen IV, tiggrin, and α- and β-integrin (Figures S3F–S3J).

The importance of integrin-mediated adhesion can be demonstrated by blocking it using RNAi. Driving the expression of RNAi constructs against the integrin subunits encoded by *mew* and *mys* or against *integrin linked kinase* prevents the establishment of the normal tip cell-alary muscle contacts, resulting in either more anterior contacts or phenotypes that resemble the loss of tip cells or alary muscle targets (cf. Figures 6U–6W with Figures 2D and 3A–3C).

The ability to form adhesive contacts with target alary muscles appears to be a property of the tip cell surface. When *cv-c* is overexpressed, tip cells develop with a greatly extended stalk (Figures 4E–4G). Where this stalk lies over an alary muscle, integrin accumulates at the site of contact, in addition to the normal contact distal to the tip cell nucleus with the adjacent, more posterior alary muscle (Figure 6X). Similarly, when *nb* is overexpressed and two tip cells develop, both accumulate integrins at the site of their more posterior alary muscle contacts (Figures 6Y–6Y′′). Increasing integrin adhesion produces a similar phenotype; in embryos homozygous for the dominantly active allele *mys*^*B51*^ (Kendall et al., 2011), tip cells remain bound to the alary muscles in A5/A6 (Figure 6Z).

## Discussion

As the embryonic renal tubules assume their mature shape they interact with other tissues, responding to Dpp guidance cues as they take up their characteristic positions in the body cavity (Bunt et al., 2010). Here we show that, in addition, a single cell at the distal end of each renal tubule makes specific transitory, and finally long-term, contacts with target tissues. These cells express a distinctive pattern of genes and show characteristic exploratory activity, which is crucial for the stereotypical looped shape and position of the tubules in the body cavity. In turn, these features have profound consequences for the efficacy of fluid homeostasis in the whole animal.

**Figure 7:**
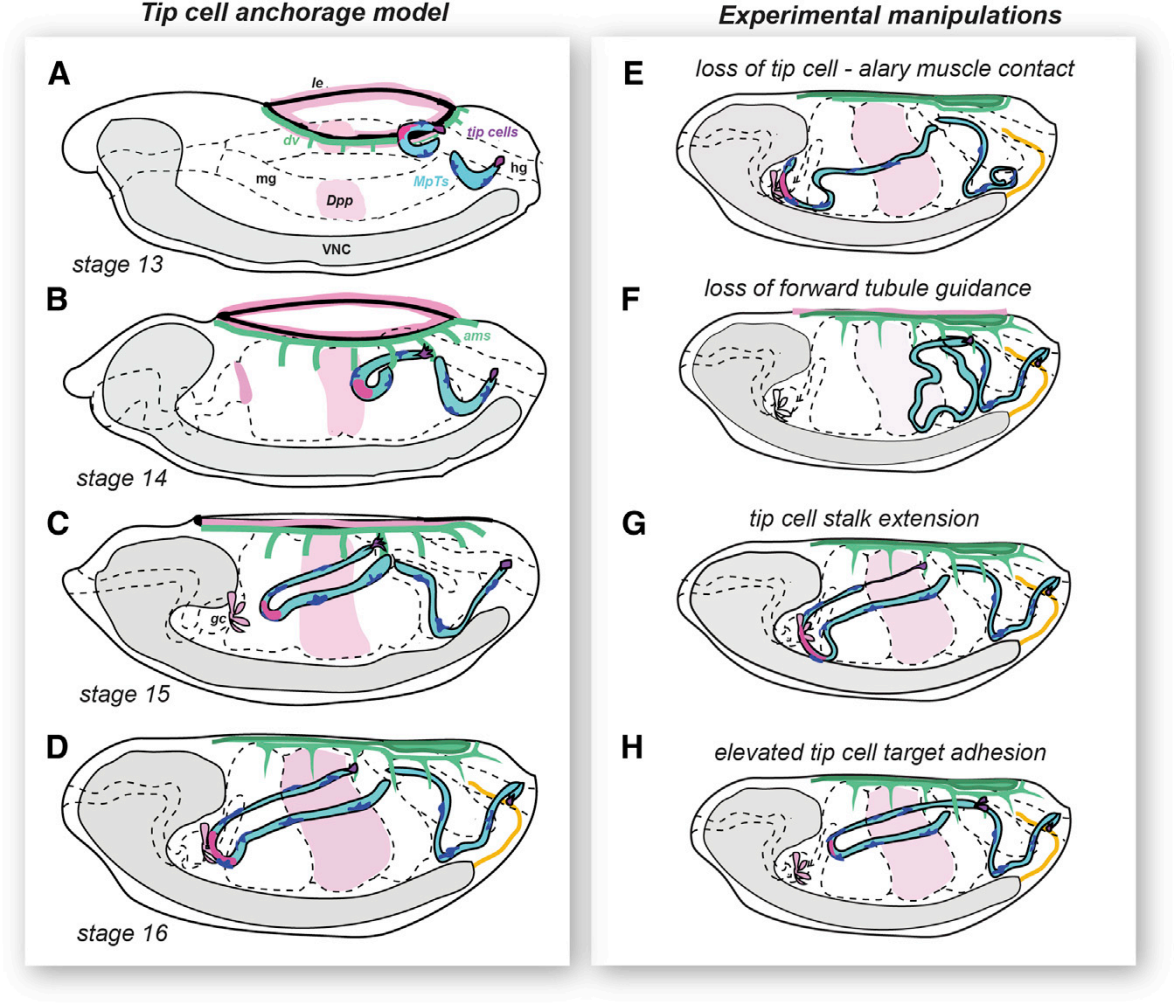
Tip-Cell-Dependent Anchorage of Anterior Tubules to Alary Muscles Elongating anterior tubules (MpTs, blue) navigate in a stereotypical manner (A–C) to achieve invariant positions by stage 16 (D). Dpp (pink) released from the leading edge (le; A), midgut (mg; A), and gastric caeca (gc; C) activate signaling (dark pink) in the tubule kink and attract the tubules forward (A–D). Distal tip cells (purple) bind to sequential alary muscles (green), first at the A5/A6 segment boundary (B) and later at A4/A5 (C) and A3/A4 (D). If tip cells or alary muscles are removed or tip cell protrusions are disrupted, anterior tubules are misshaped and mispositioned, with shorter distal ends intimately associated with the gastric caeca (E). Final tubule architecture reflects a balance between the strength of Dpp-guided anterior attraction and antagonistic tip cell anchorage. If Dpp signaling is abrogated, tubules stall posteriorly with tip cells bound to A5/A6 targets (F). Conversely, if tip cell-muscle adhesion strength is increased (by increasing tip cell number or adhesion; H) tubules remain posterior, with tip cells bound to A5/A6 or A4/A5 targets. Tip cell RhoGTPase activity must be strictly controlled; inhibition of Rho1 lengthens the tip cell stalk, and tubules move further anteroventrally (G). ams, alary muscles; hg, hind gut; VNC, ventral nerve cord.

We suggest that the elongation and forward extension of the tubules result from the combined effects of cell rearrangements that lengthen the tubule and the response of kink region cells to regional Dpp guidance cues (Figures 7A–7D). Our evidence indicates that the tip cells act as anchors, through their interactions with alary muscles, so that tubules are tethered at both ends, the proximal end being attached through ureters to the hindgut. These attachments perform two functions: they stabilize the looped architecture, maintaining the kink close to the tubule midpoint, and they limit forward and ventral movement to ensure the stereotypical tubule arrangement in the body cavity.

If tip cell contact with the alary muscles is lost, the kink “unravels,” shifting distalward, and the tubule as a whole extends too far into the anterior, with the distal region lying more ventrally close to the Dpp-expressing gastric caeca (Figure 7E). Confirming the existence of a forward tractive force responsible for tip cell detachment are the distortion of transient alary muscle targets before the tip cell detaches (Movie S2) and the characteristic “recoil” seen when the tip cell is ablated (Movies S4 and S5). Evidence that this results from the response to guidance cues is the failure of tip cells to detach from their first alary muscle contact (A5/A6) in the absence of the midgut Dpp guidance cue (Figure 7F) and the more anterior location of the kink region (close to the gastric caeca) in tubules where the tip cell stalk is greatly extended, for example when the activity of RhoA is repressed (Figure 7G). The critical nature of the balance between these forward and restraining influences is also revealed when adhesion between the tip cells and alary muscles is increased by manipulating tip cell number or adhesive strength (Figure 7H). In each case, the tip cells remain attached to alary muscles posterior to their normal final contacts, and this results in more posterior positioning of the whole tubule. Together, these results strongly suggest that tip cells detach because the forward movement of tubules overcomes the adhesive strength of their early transient contacts.

The final tip cell/alary muscle target is highly reproducible, suggesting recognition through segmental identity, the A3/A4 target being the first encountered by the tip cell that expresses *Ubx* (LaBeau et al., 2009). However, altering *Ubx* expression in alary muscles has no effect on the final tip cell contact. Instead, it appears that tip cells adhere to each alary muscle they contact, and the final target depends on the balance between forward tubule movement and the strength of tip cell/target adhesion (Figure 7). Consistent with this view, when all the normal muscle targets are ablated, tip cells can make stable contacts with the A2/A3 alary muscle.

From the time that they are specified, tip cells show distinctive patterns of gene expression, morphology, and behavior critical to their ability to make alary muscle contacts; they form dynamic filopodia, which explore the alary muscle surface, remain denuded of the BM that envelops the rest of the tubule, and express cell-adhesion proteins, including Nrm and integrins. Protrusive activity depends on the rapid turnover of actin, mediated by regulators such as Rac GTPase and the actin-capping protein Enabled, which are active in tip cells.

BM deposition basally around the tip cells severely inhibits protrusive behavior, and tip cells therefore employ multiple mechanisms to ensure that they remain denuded. These include the absence of expression of factors that promote BM deposition and stabilization (hemocyte attractants, BM components, or receptors), the removal by transcytosis of any BM that is deposited, and expression of MMP1, which is able to cleave matrix components (Gross and Lapiere, 1962). MMP1 is expressed late during tubule elongation and the protein is localized apically in tip cells (H.W., unpublished data), suggesting that its function might be to degrade transcytosed BM proteins.

Protrusive exploratory behavior results in adhesive contacts made possible through tip cell expression of Nrm and integrins. Nrm is a homophilic cell-adhesion molecule of the Ig-domain superfamily (Kania et al., 1993). The binding partner for tip cell Nrm is unclear, as alary muscles do not express it. However, driving *nrm* expression in alary muscles induces strong adhesion, resulting in tip cells remaining bound to their first target in A5/A6. It is possible that Nrm in tip cells normally makes heterophilic associations with Ig domain-containing proteins such as Dumbfounded (Kirre), which is expressed in alary muscles and is sufficient, when overexpressed, to induce more posterior target adhesion (H.W., unpublished data).

Tip cells express integrins, and complexes accumulate as each target contact is made, but initially they do not lead to long-term adhesion. We suggest that the strength of adhesion increases with successive contacts, either through increased expression of integrins and their associated factors or by regulated adhesive complex turnover, as shown in other tissues (Ulrich and Heisenberg, 2009; Webb et al., 2002). Once the final tip cell contact is made, BM accumulates around the tip cell-alary muscle surface, increasing the concentration of integrin ligands at the junction. The accompanying decline in the protrusive activity of the tip cell could also result from integrin-mediated adhesion, which is known to reduce levels of the actin-capping protein Enabled (Delon and Brown, 2009). This sequence of events parallels the mechanism by which elongated myotubes and tendon cells establish their myotendinous junctions (Martin-Bermudo et al., 1998).

Once the anterior tubule tip cells make their final alary muscle contact, they remain attached throughout development into adult life. Such interaction of excretory tubule tips with muscles is a common feature of renal systems in insects, either with alary muscles or with fine striated muscles that spiral along the tubule (see Wigglesworth, 1972). Muscle contacts increase tubule movement, maximizing the effectiveness of excretion, by increasing hemolymph sampling and enhancing tubule flow. Similar contacts are found outside the arthropods; the flame cells that cap planarian protonephridial tubes develop prominent filopodia and interact with nearby muscle fibers (McKanna, 1968), providing anchorage, thought to be important during branching morphogenesis in this system (Rink et al., 2011).

Tip cells or groups of cells at the distal tips of outgrowing epithelial tubes act as organizers in tubular systems, from the migrating *Dictyostelium* slug to the branching epithelial scaffolds of human organs. As in fly renal tubules, these distinctive cells regulate cell division (Kimble and White, 1981) and guided tubule extension (e.g., Caussinus et al., 2008; Bradley et al., 2003; Blelloch et al., 1999; Su et al., 2000), and in mammalian systems they control branching morphogenesis (Gerhardt et al., 2003; Watanabe and Costantini, 2004; Chi et al., 2009).

However, in distinct contrast to the role of tip cells in the morphogenesis of these systems, the tip cells of the anterior renal tubules play no role in *leading* outgrowth. Instead, they act to counteract outgrowth, and importantly this leads to the development of a looped tubular structure both by tethering the distal tips of tubules close to their proximal junction with the ureter and by maintaining the tightness of the tubule kink region. Looped tubular structures are relatively uncommon; a tubule tree as in the lung, pancreas, or liver or an anastomosing network as in the vascular system is more frequently seen. However, a striking example of looped tubules is found in the mammalian kidney, where the distal and proximal convoluted tubules together with the loop of Henle connect the tubule tip (at the glomerulus) to the collecting duct (close to the site of urine outflow). Looping of both the nephron and its vascular supply creates a countercurrent system that maximizes the efficiency of ion and fluid homeostasis. Such exchange systems also occur in insects with specialized diets (e.g., Le Caherec et al., 1997) or those living in dry conditions (Ramsay, 1964). Countercurrent exchange has not been demonstrated in *Drosophila melanogaster* tubules, where it is more likely that the looped tubule structure is important for effective hemolymph sampling.

In the development of the mammalian nephron, as in fly renal tubules, both the site of connection to the ureter and the tubule tip, the renal corpuscle, are established early in organ development (Potter, 1972; Georgas et al., 2009; Cho and Dressler, 2003) so that tubule extension, by both cell proliferation (Fischer et al., 2006) and rearrangements (Karner et al., 2009), occurs between these fixed points. It will be interesting to discover whether similar tissue interactions stabilize the position of the developing glomerulus, and so play a prominent role in maintaining the looped structure as kidney tubules extend, resulting in the final intricate and regular array of nephrons apparent in the mature mammalian kidney.

## Experimental Procedures

### *Drosophila* Genetics

Fly stocks were maintained according to standard protocols (Greenspan, 1997). Crosses were carried out at 25°C unless otherwise stated. For strains, see the Supplemental Experimental Procedures.

### Immunostaining and In Situ Hybridization

Immunostaining was performed using standard techniques with the antibodies listed (see Supplemental Experimental Procedures). An amplification step using streptavidin-conjugated FITC, Cy3, or Cy5 was performed when required.

RNA localization was performed by in situ hybridization using DIG-labeled RNA probes generated by in vitro transcription from DNA templates (see Supplemental Experimental Procedures). Hybridization and staining were done according to standard protocols (Nagaso et al., 2001; Tautz and Pfeifle, 1989).

### Live Imaging

Staged embryos were dechorionated in bleach and mounted in Voltalef 10S oil with a raised coverslip. Live imaging was performed using a Leica SP5 scanning laser microscope. To photoconvert Kaede, cells were exposed to a 55–85 s UV pulse using FRAP software on the Leica SP5 system.

### Laser Ablation

Dechorionated embryos were mounted with Scotch double-sided tape in PBS solution. Cell ablation was performed using a 63× water-dipping lens (N.A. 0.9) on a Yokogawa spinning disk (CSU-10) confocal microscope fitted with a pulsed nitrogen laser (MicroPoint). Image acquisition and microscope control were by MetaMorph (version 7.0) software (Molecular Devices).

## Supplemental Information

Document S1. Supplemental Experimental Procedures, Figures S1–S3, and Table S1

Movie S1. Tip Cells Exhibit Dynamic Protrusive Membrane Activity, Related to Figure 1 Anterior tip cells extend and retract membrane protrusions (arrows). Stage 15 tubules express membrane CD8-GFP driven by *ctB-Gal4*. Images were taken at 60 s intervals.

Movie S2. Anterior Tip Cells Interact with Successive Alary Muscle Targets, Related to Figure 1 Tip cell loses contact with the A4/A5 alary muscle (am) and forms a new contact with the more anterior A3/A4 alary muscle (arrows). Tip cell dynamic protrusive behavior correlates with alary muscle interaction. *ctB-Gal4* and *tup-Gal4* drive membrane CD8-GFP expression in tubule cells (MpT) and muscles, respectively. Images were taken at 60 s intervals. A z stack maximal projection is shown.

Movie S3. Anterior Tip Cells Interact with Successive Alary Muscle Targets, Related to Figure 1 Tip cell loses contact with the A4/A5 alary muscle (am) and forms a new contact with the A3/A4 alary muscle (arrows); tip cells display dynamic protrusive behavior on alary muscle interaction. *ctB-Gal4* and *tup-Gal4* drive membrane CD8-GFP expression in tubule cells (MpT) and muscles, respectively. Images were taken at 60 s intervals. Single z sections through the z stack are shown.

Movie S4. Laser Ablation of Anterior Tip Cells, Related to Figure 2 Laser ablation of a stage 13 anterior tip cell (arrow) expressing membrane CD8-GFP driven by *ctB-Gal4*. The tubule “recoils” anteriorly immediately after tip cell ablation.

Movie S5. Tip Cell Ablation Perturbs Anterior Tubule Shape and Position, Related to Figure 2 Anterior tubules (*ctB-Gal4>UAS-mCD8-GFP*) lose their stereotypical shape after tip cell ablation; the distal tubule ends move further anteroventrally and the kink region shifts distally. Images were taken every 3 min.

Movie S6. Laser Ablation of Embryonic Alary Muscles, Related to Figure 3 Alary muscle ablation (A4/A5; membrane CD8-GFP driven by *tup-Gal4*) in a stage 14 embryo.
