# Supplementary material for: Tip Cells Act as Dynamic Cellular Anchors in the Morphogenesis of Looped Renal Tubules in Drosophila
Source: Dev Cell. 2013 Nov 11;27(3):331–44. doi: 10.1016/j.devcel.2013.09.020 (PMC3898071; doi:10.1016/j.devcel.2013.09.020)
Supplement: Document S1. Supplemental Experimental Procedures, Figures S1–S3, and Table S1 [file mmc1.pdf]

Developmental Cell, Volume *27*

Supplemental Information

Tip Cells Act as Dynamic Cellular Anchors in the Morphogenesis of Looped Renal Tubules in *Drosophila*

Helen Weavers and Helen Skaer

Inventory of Supplemental Information

Figure S1 (related to Figure 2)
Figure S2 (related to Figure 3)
Figure S3 (related to Figure 6)

Supplemental Figure Legends S1 – S3

Movie S1 (related to Figure 1)
Movie S2 (related to Figure 1)
Movie S3 (related to Figure 1)
Movie S4 (related to Figure 2)
Movie S5 (related to Figure 2)
Movie S6 (related to Figure 3)

Supplemental Movie Legends S1 – S6

Supplemental Table S1 (related to Figures 2 & 4)

Supplemental Experimental Procedures

Supplemental References

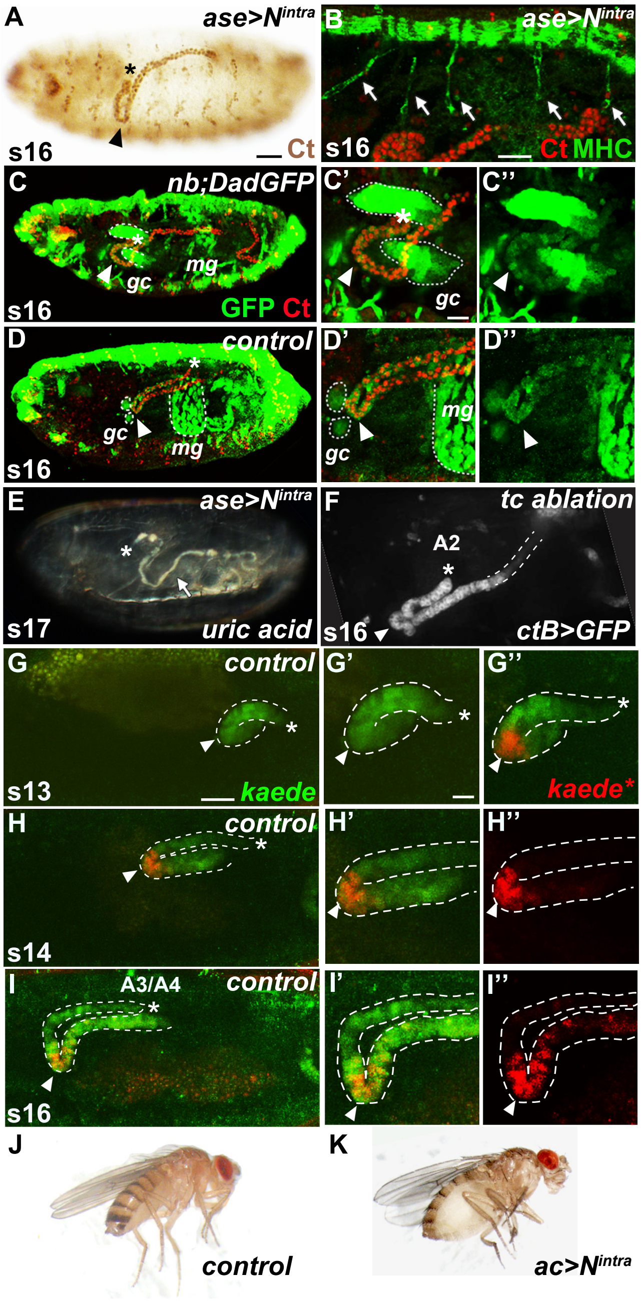
A
ase>N^intra
s16
Ct
B
ase>N^intra
s16
Ct MHC
C
nb;DadGFP
gc
mg
s16
GFP Ct
C'
gc
C''
D
control
gc
mg
s16
D'
mg
gc
D''
E
ase>N^intra
s17
uric acid
F
tc ablation
A2
s16
ctB>GFP
G
control
s13
kaede
G'
G''
kaede*
H
control
s14
H'
H''
I
A3/A4
control
s16
I'
I''
J
control
K
ac>N^intra

Figure S1

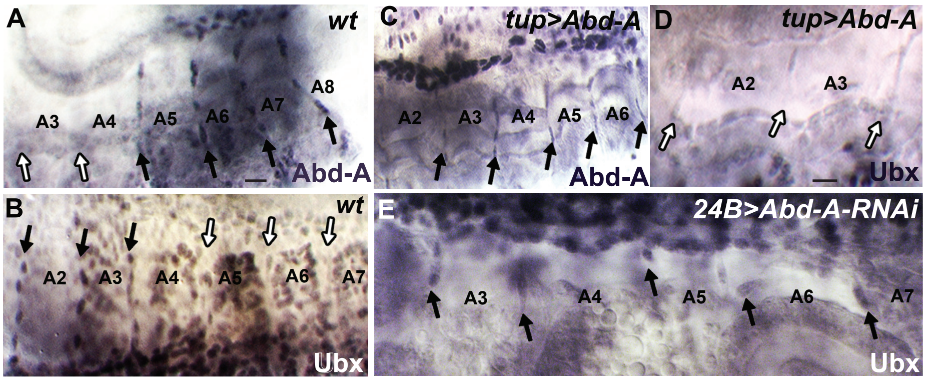
A
wt
A3
A4
A5
A6
A7
A8
Abd-A
C
tup>Abd-A
A2
A3
A4
A5
A6
Abd-A
D
tup>Abd-A
A2
A3
Ubx
B
wt
A2
A3
A4
A5
A6
A7
Ubx
E
24B>Abd-A-RNAi
A3
A4
A5
A6
A7
Ubx

Figure S2

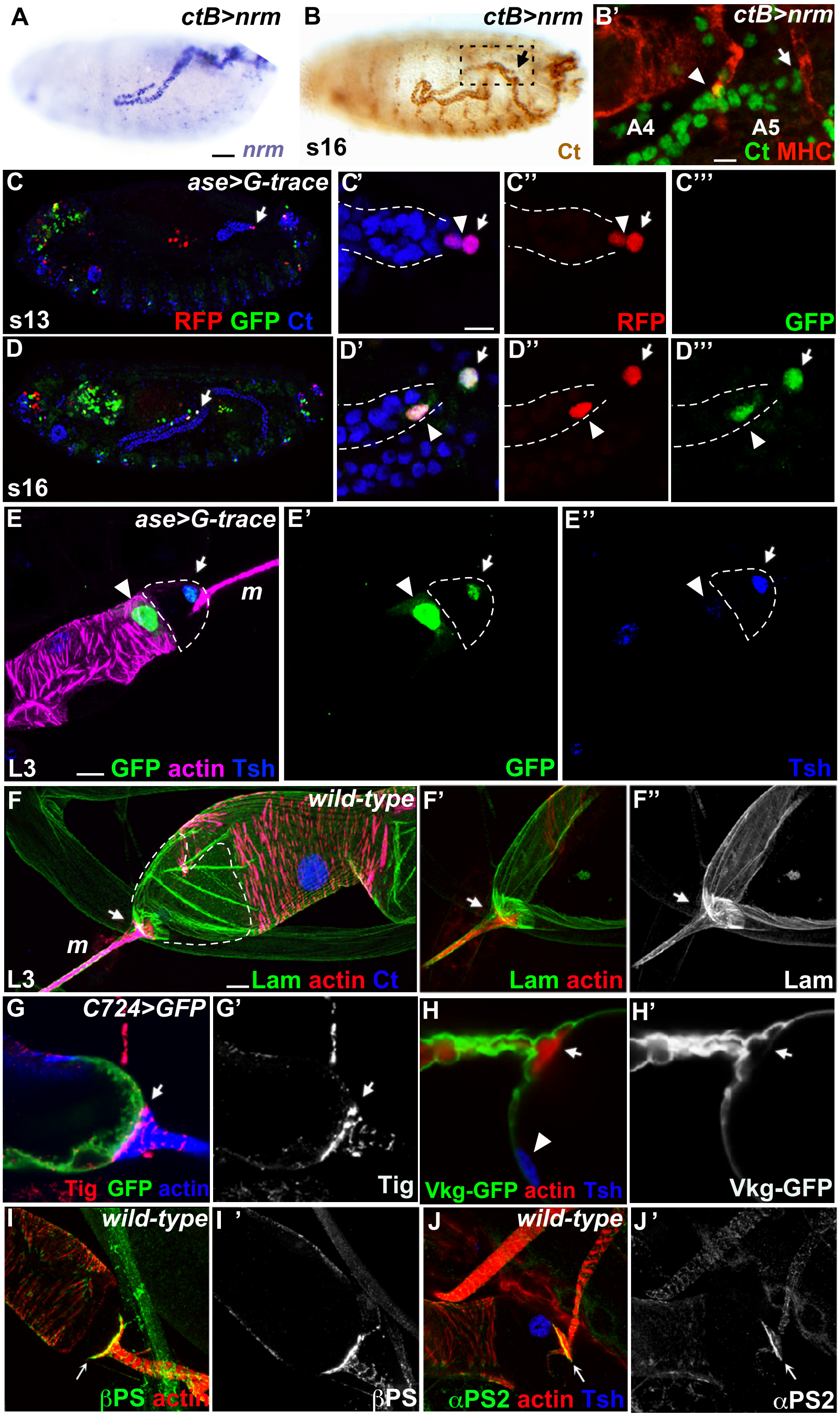
A
ctB>nrm
nrm
B
ctB>nrm
s16
Ct
B'
ctB>nrm
A4
A5
Ct MHC
C
ase>G-trace
s13
RFP GFP Ct
C'
C''
RFP
C'''
GFP
D
s16
D'
D''
D'''
E
ase>G-trace
m
L3
GFP actin Tsh
E'
GFP
E''
Tsh
F
wild-type
m
L3
Lam actin Ct
F'
Lam actin
F''
Lam
G
C724>GFP
Tig GFP actin
G'
Tig
H
Vkg-GFP actin Tsh
H'
Vkg-GFP
I
wild-type
βPS actin
I'
βPS
J
wild-type
αPS2 actin Tsh
J'
αPS2

Figure S3

<u>**Supplemental Figure Legends**</u>

Figure S1 (related to Figure 2)

Tip cell removal perturbs tubule shape and positioning but not Dpp sensitivity or uric acid formation.

(A) Tip cell removal (*aseGal4>UAS-N*^{intra}) results in the same tubule (Ct, brown) positioning defects as seen in *nb* mutants. Distal tubule ends move further anteroventrally (asterisk) and the kink shifts distally (arrowhead).

(B) Tip cell removal does not perturb the number or arrangement of embryonic alary muscles (MHC, green, arrows). A mispositioned anterior tubule is labelled by Ct (red).

(C-D) Dpp signalling is active (shown by Dad expression; DadGFP, green) in the kink region (arrowheads) of *nb* mutant tubules (Ct, red) lacking tip cells (C', asterisks) as in control tubules (D). The entire distal and kink regions of *nb* tubules (C) are associated with the Dpp-expressing gastric caeca (gc) but only the kink region of control tubules (D).

(E) Tubules lacking tip cells (asterisk) accumulate uric acid (white, arrow) by the end of embryogenesis despite being mispositioned in the body cavity.

(F) Tip cell ablation later in development at stage 14 perturbs tubule arrangement (*ctBGal4>UASmCD8GFP*). The distal region (asterisk) moves further anteroventrally and the kink moves distally (arrowhead).

(G-I) The kink region of stage 13 control tubules expressing the photoconvertible fluorophore Kaede (green) labelled by local Kaede activation (Kaede*, red, arrowhead); the majority of labelled cells remain in the kink region throughout tubule elongation and navigation (H-I - H"-I", arrowheads).

(J-K) Control (J) and tip cell-less (K) one week old adults. Adult flies lacking tip cells have grossly distended abdomens.

Scale bars: 50μm A, C-F; 30μm B, G-I; 10μm C'-D', G'-I'.

Figure S2 (related to Figure 3)

Manipulation of alary muscle segmental identity.

(A-B) Four posterior-most alary muscle pairs (A4/A5 to A7/A8) express *Abd-A* (A, black arrows) but not *Ubx* (B, white arrows). Three anterior-most alary muscle pairs (A1/A2 to A3/A4) express *Ubx* (B, black arrows) but not *Abd-A* (A, white arrows).

(C-D) Manipulation of alary muscle A-P identity does not affect target choice. Ectopic Abd-A (driven by *tupGal4*) 'posteriorises' all alary muscles with loss of Ubx (D, white arrows) and gain of Abd-A (C, black arrows).

(E) *24BGal4>Abd-A-RNAi* 'anteriorises' alary muscles, which gain Ubx (arrows).

Scale bars: 10μm A-C; 5μm D-E.

Figure S3 (related to Figure 6)

Tip cells persist and remain bound in an integrin-associated manner to alary muscle targets.

(A-B) Ectopic *nrm* expression driven throughout tubules by *ctB-Gal4* (*in situ* blue, A); tubules (Ct, brown, B; green, B') stall in the posterior with tubule cells (arrowhead) as well as tip cells (arrow) associated with alary muscles (MHC, red).

(C-E) Expression of G-TRACE (Evans et al, 2009; *aseGal4>UAS-RFP,UASflp1,Ubi-p63E(FRT.stop.FRT)eGFP*) in the tip cell lineage traces tip cells from embryo (C-D) to larvae (E). RFP (red, C-E) gives a real-time readout of *Gal4* expression, labelling embryonic tip (arrows) and sibling cells (arrowheads, tubules Ct, blue). GFP (green) is stably expressed in the tip cell lineage and labels tip and sibling cells in 3rd instar larval tubules (E). Larval tip cell also express *teashirt* (Tsh, blue, E).

(F-J) The ECM proteins Laminin (green, F-F', white, F''), Tiggrin (red, G, white, G'), the integrin subunits βPS (green, I, white, I') and αPS2 (green, J, white, J') localise to the tip cell-alary muscle junction of 3rd instar larvae (arrows) but the Collagen IV Viking (green, H, white, H'; arrowhead labels tip cell nucleus) does not. Phalloidin labels tip cell and muscle

actin (red, F, H-J and blue, G). Z-projections (F-F'', I-I' and J-J') and single sections (G-G' and H-H').

Scale bars: 50μm A-D; 10μm B'-D''', E-E''; 5μm F-J'.

Movie S1 (related to Figure 1)

Tip cells exhibit dynamic protrusive membrane activity.

Anterior tip cells extend and retract membrane protrusions (arrows). Stage 15 tubules expressing membrane CD8-GFP driven by *ctB-Gal4*. Images taken at 60 second intervals.

Movies S2 and S3 (related to Figure 1)

Anterior tip cells interact with successive alary muscle targets.

Tip cell loses contact with the A4/A5 alary muscle (am) and forms a new contact with the more anterior A3/A4 alary muscle (arrows). Tip cell dynamic protrusive behaviour correlates with alary muscle interaction. *ctB-Gal4* and *tup-Gal4* drive membrane CD8-GFP expression in tubule cells (MpT) and muscles, respectively. Images taken at 60 second intervals. Z-stack maximal projection in Movie S2 and single z-sections in Movie S3.

Movie S4 (related to Figure 2)

Laser ablation of anterior tip cells.

Laser ablation of stage 13 anterior tip cell (arrow) expressing membrane CD8-GFP driven by *ctB-Gal4*. The tubule 'recoils' anteriorly immediately after tip cell ablation.

Movie S5 (related to Figure 2)

Tip cell ablation perturbs anterior tubule shape and position.

Anterior tubules (*ctB-Gal4>UAS-mCD8-GFP)* lose their stereotypical shape after tip cell ablation; distal tubule ends move further anteroventrally and the kink region shifts distally. Images taken every 3 minutes.

Movie S6 (related to Figure 3)

Laser ablation of embryonic alary muscles.

Alary muscle ablation (A4/A5; membrane CD8-GFP driven by *tup-Gal4*) in a stage 14 embryo.

Supplemental Table S1 (related to Figures 2 and 4)

Analysis of kink position along the proximal-distal tubule axis following tip cell removal or cytoskeletal modification

	**(A) Cell number (% total tubule)**		**(B) Length as % of total tubule**	
	distal	proximal	distal	proximal
**Wild type**	60±1.6 (42.6)	81±1.6 (57.4) (n=5)	44.8%±1.6	55.2%±1.6 (n=6)
***nb*^{1} mutant**	39±4.0 (29.3)	94±5.9 (70.7) (n=6)	25.3%±4.9	74.7%±4.9 (n=4)
ase>cv-c	39±1 (29.5)	93±3.8 (70.5) (n=3)	28.5%±5.1	71.5%±5.1 (n=4)
ase>FP4-mito	35±7.5 (25.6)	102±7.2 (74.5) (n=5)	29.4%±4.0	70.6%±4.0 (n=4)

The relative position of the kink region along the proximo-distal axis of anterior tubules by (A) counting the number of tubule cells in the distal (tip cell to kink) and proximal (kink to ureter) regions of fully elongated stage 16 anterior tubules and (B) measuring the relative lengths of distal (excluding the tip cells) and proximal tubule regions in ImageJ. Analyses were performed on wild-type, *nb*^{1} mutant (tip cell loss), *ase>cv-c* (tip cell elongation) and *ase>FP4mito* (inhibition of tip cell protrusive activity) embryos. Figures are given ± SD.

Supplemental Experimental Procedures

Drosophila Genetics

Wild-type analysis was performed on *OregonR* embryos. The following alleles were used for mutant analysis: *numb*^{1}, *Ubx*$^{9.22}$, *dsparc*^{136} (all from Bloomington Stock Centre, Indiana), *mys*^{active} (gift of N. Brown). *FM7c,ftz-lacZ, CyO,wg-lacZ* and *TM3,ftz-lacZ* blue balancers were used to identify homozygous embryos except for *Ubx*$^{9.22}$ (balanced over TM1) where a homeotic transformation which results in multiple anterior spiracles was used to positively identify homozygous embryos when stained with an antibody against Cut. The GAL4 system (Brand and Perrimon, 1993) was used for mis-expression experiments with the following lines: *byn-Gal4* (gift of R. Reuter), *ctB-Gal4* (gift of V. Sudarsan), *ac-Gal4* (gift of P. Simpson), *tup-Gal4* (gift of J. Navascués), *C724-Gal4* (gift of J. Dow), *ase-Gal4, 24B-Gal4* (Bloomington), *UAS-Abd-A* (gift of I. Miguel-Aliaga), *UAS-Abd-A-RNAi* (gift of L. Perrin), *UAS-cv-c* (gift of B. Denholm), *UAS-Dg* (gift of W.M. Deng), *UAS-FP4mito* (gift of J. Casanova), *UAS-kaede* (gift of W. Grueber), *UAS-Rab5*^{DN,GFP}, *UAS-cd8-gfp, UAS-nrm, UAS-Rho*^{N19}, *UAS-Rac*^{N17}, *UAS-numb, UAS-N*^{act}, *UAS-tauGFP, UAS-G-trace* (all Bloomington), *UAS-nrm-RNAi, UAS-mys-RNAi, UAS-ilk-RNAi, UAS-mew-RNAi* (all VDRC). The following reporter lines were used: *dad-gfp(nuclear)* (gift of M. Affolter), *islet-taumycGFP* (gift of S. Thor), *vkg-gfp* (gift of W. Chia), *nrm-lacZ, CtB-eGFP* (gift of K. Campbell).

Immunohistochemistry, *in situ* hybridisation and uric acid visualisation

For immunostaining the following antibodies were used: mouse anti-Ct (1:200, DSHB), goat anti-GFP (1:500, Abcam,), rabbit anti-βgalactosidase (1:10000, Cappel), rabbit anti-MHC (1:500, D. Kiehart), rat anti-Abd-A (1:250, I. Miguel-Aliaga), mouse anti-Ubx (1:5, DSHB), rabbit anti-Tsh (1:3000, L. Fasano), mouse anti-Futsch (1:200, DSHB), mouse anti-Dlg (1:500, DSHB), rabbit anti-Dg (1:200, D. Bergstralh), mouse anti-Tiggrin (1:1000, J. Fessler), mouse anti-Ena (1:500, DSHB), rabbit anti-Pcan (1:1000, S. Baumgartner), rabbit anti-Baz

(1:1500, A. Wodarz), mouse anti-PS2 (1:10, DSHB), mouse anti-βPS (1:10, DSHB), mouse anti-Talin (1:10, DSHB) and rabbit anti-Laminin (1:1000, J. Fessler). Biotinylated secondary antibodies were used in conjunction with the Vector Elite ABC kit (Vector Laboratories) for DAB staining or fluorescent secondary antibodies conjugated to FITC or Cy3 (1:200, Jackson ImmunoResearch).

Embryos were collected from agar apple juice plates, dechorionated in bleach, fixed for 20 minutes in 4% paraformaldehyde, and de-vitellinised in a 1:1 heptane:methanol mixture by vigorous shaking. Microtubules were visualized by incubating embryos in FITC-conjugated mouse anti-Tubulin (1:200, Sigma). Actin was visualized by incubating dissected larval preparations in rhodamine-conjugated Phalloidin (Sigma) diluted 1:20 in PBS-TX-BSA for two hours. Third instar larvae were dissected in PBS solution on Sylgard plates using insect pins and microscissors. Dissected larvae were fixed in 4% paraformaldehyde for 15 minutes before washing in PBS-TX-BSA and subsequent immunostaining performed as described above for embryos.

RNA probes for *in situ* hybridization were made from cDNA obtained from the *Drosophila* Genome Resource Centre for the following genes: *mmp1* (RE19818), *nrm* (IP03054), *dg* (GH09323). Probes for *pvf1-3, lanA* and *lanB2* were kindly provided by Stephanie Bunt (Bunt et al., 2010). For light microscope analysis, embryos were either dehydrated and cleared in Histoclear (Fisher-Scientific) before mounting in DPX (BDH Laboratory Supplies), or alternatively mounted in 80% glycerol to facilitate embryo rolling, and then imaged using a Zeiss Axioplan with a JCB KY-F55B digital camera. For fluorescent analysis, all embryos were mounted in Vectashield (Vector Laboratories) and imaged using a Leica SP5 scanning confocal microscope and LSM software. For live imaging, time-lapse series were assembled using ImageJ imaging software (NIH).

Uric acid was visualised in stage 17 embryos mounted in water under phase contrast with polarised light.

Supplemental References

Brand, A. H., and Perrimon, N. (1993). Targeted gene expression as a means of altering cell fates and generating dominant phenotypes. Development *118*, 401-415.

Evans, C., Olson, J., Ngo, K., Kim, E., Lee, N., Kuoy, E., Patananan, A., Sitz, D., Tran, P., Do, M., et al. (2009). G-TRACE: rapid Gal4-based cell lineage analysis in Drosophila. Nature Methods *6*, 603–605.
