# Supplementary figures and images for: Tip Cells Act as Dynamic Cellular Anchors in the Morphogenesis of Looped Renal Tubules in Drosophila
Source: Dev Cell. 2013 Nov 11;27(3):331–44. doi: 10.1016/j.devcel.2013.09.020 (PMC3898071; doi:10.1016/j.devcel.2013.09.020)

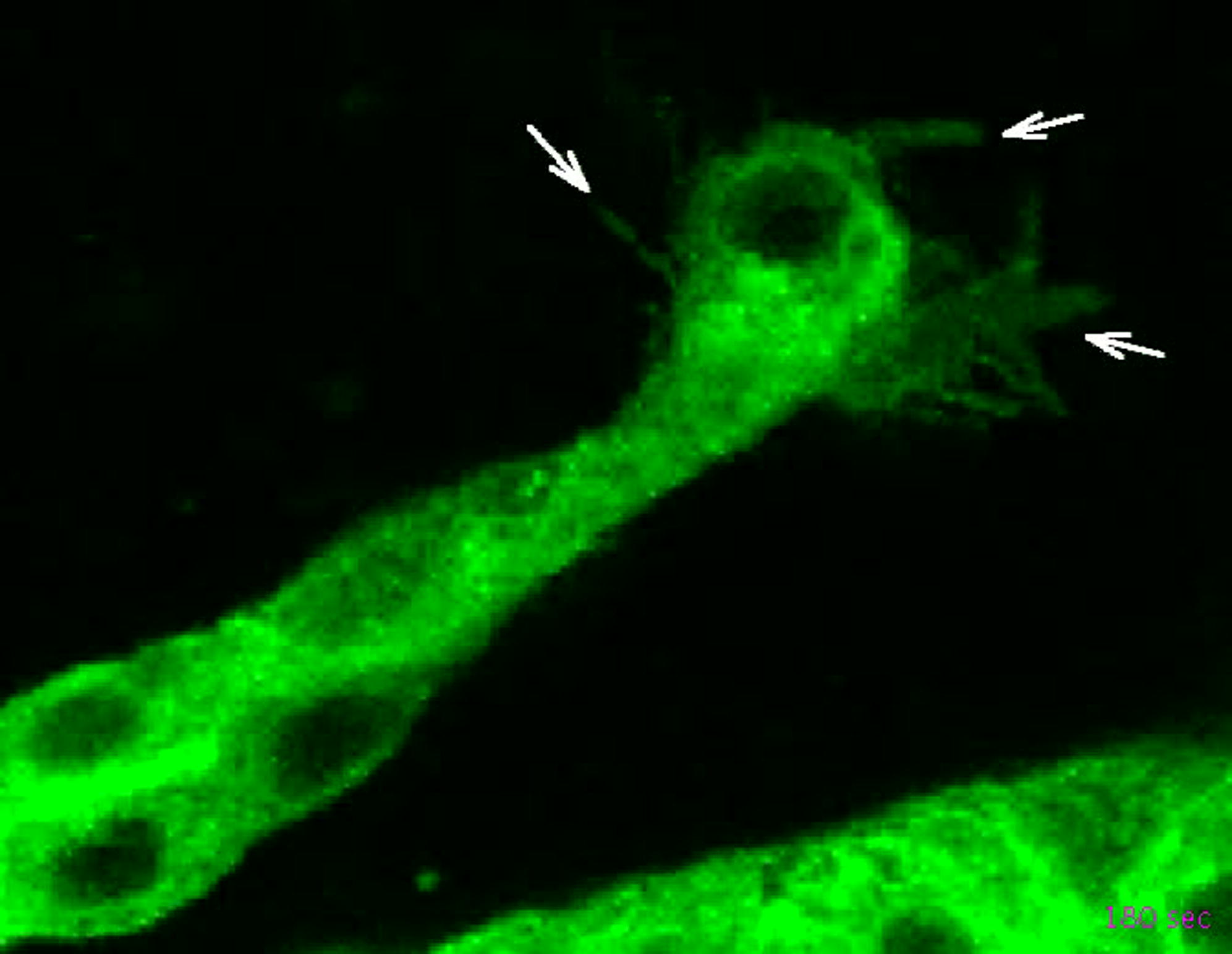

Supplement: Movie S1. Tip Cells Exhibit Dynamic Protrusive Membrane Activity, Related to Figure 1 — Anterior tip cells extend and retract membrane protrusions (arrows). Stage 15 tubules express membrane CD8-GFP driven by ctB-Gal4. Images were taken at 60 s intervals. [file mmc2.jpg]

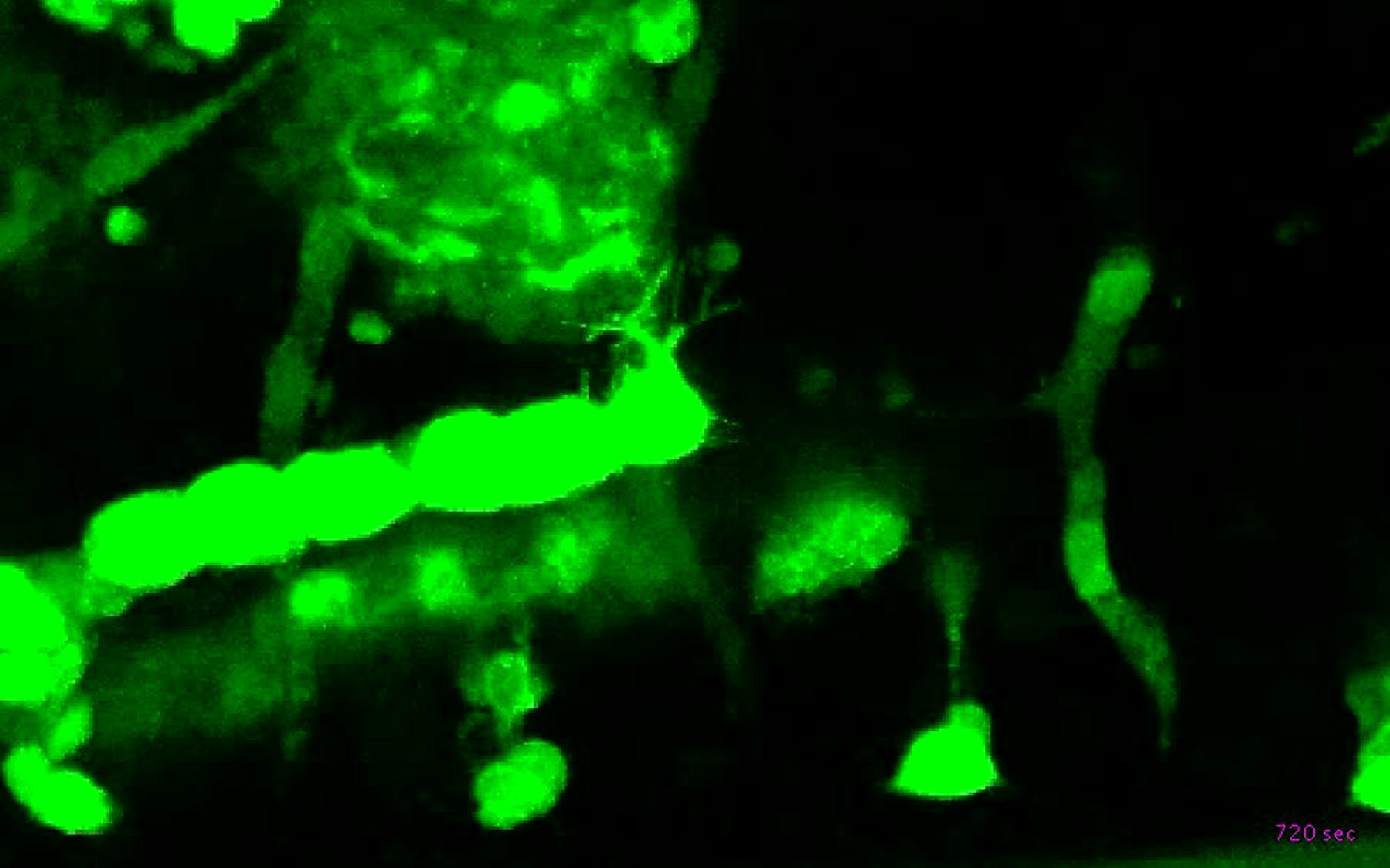

Supplement: Movie S2. Anterior Tip Cells Interact with Successive Alary Muscle Targets, Related to Figure 1 — Tip cell loses contact with the A4/A5 alary muscle (am) and forms a new contact with the more anterior A3/A4 alary muscle (arrows). Tip cell dynamic protrusive behavior correlates with alary muscle interaction. ctB-Gal4 and tup-Gal4 drive membrane CD8-GFP expression in tubule cells (MpT) and muscles, respectively. Images were taken at 60 s intervals. A z stack maximal projection is shown. [file mmc3.jpg]

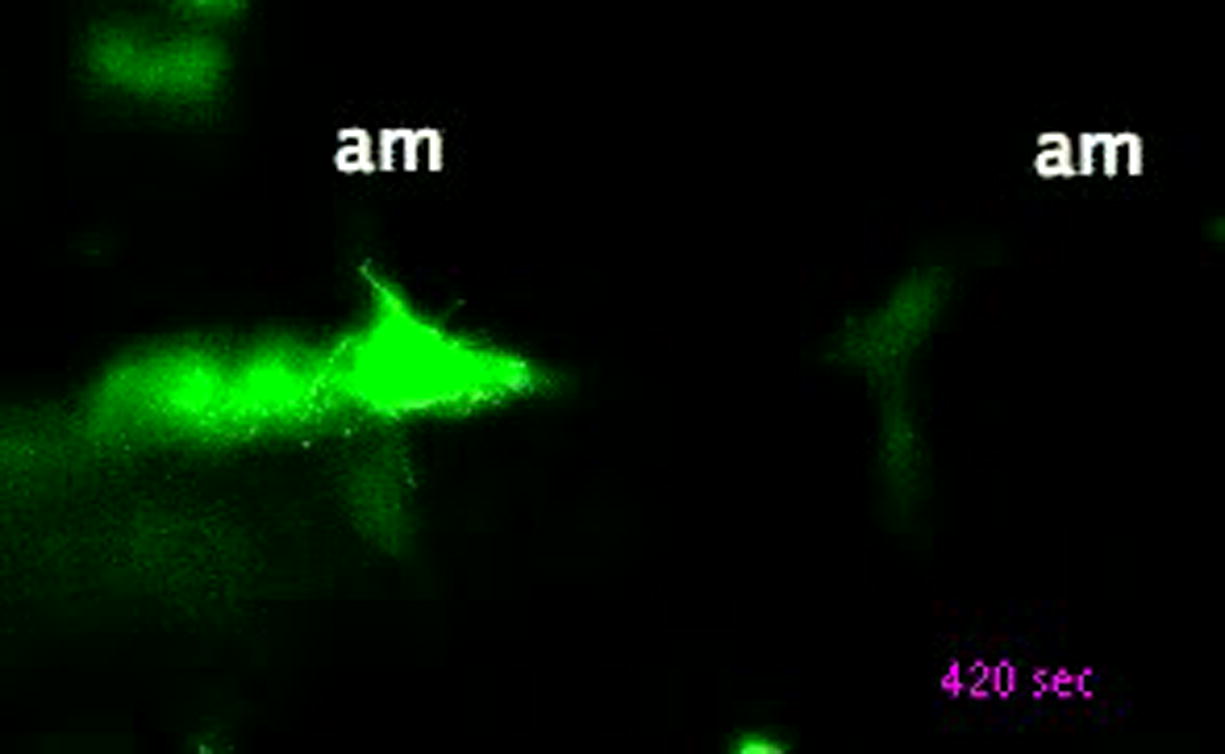

Supplement: Movie S3. Anterior Tip Cells Interact with Successive Alary Muscle Targets, Related to Figure 1 — Tip cell loses contact with the A4/A5 alary muscle (am) and forms a new contact with the A3/A4 alary muscle (arrows); tip cells display dynamic protrusive behavior on alary muscle interaction. ctB-Gal4 and tup-Gal4 drive membrane CD8-GFP expression in tubule cells (MpT) and muscles, respectively. Images were taken at 60 s intervals. Single z sections through the z stack are shown. [file mmc4.jpg]

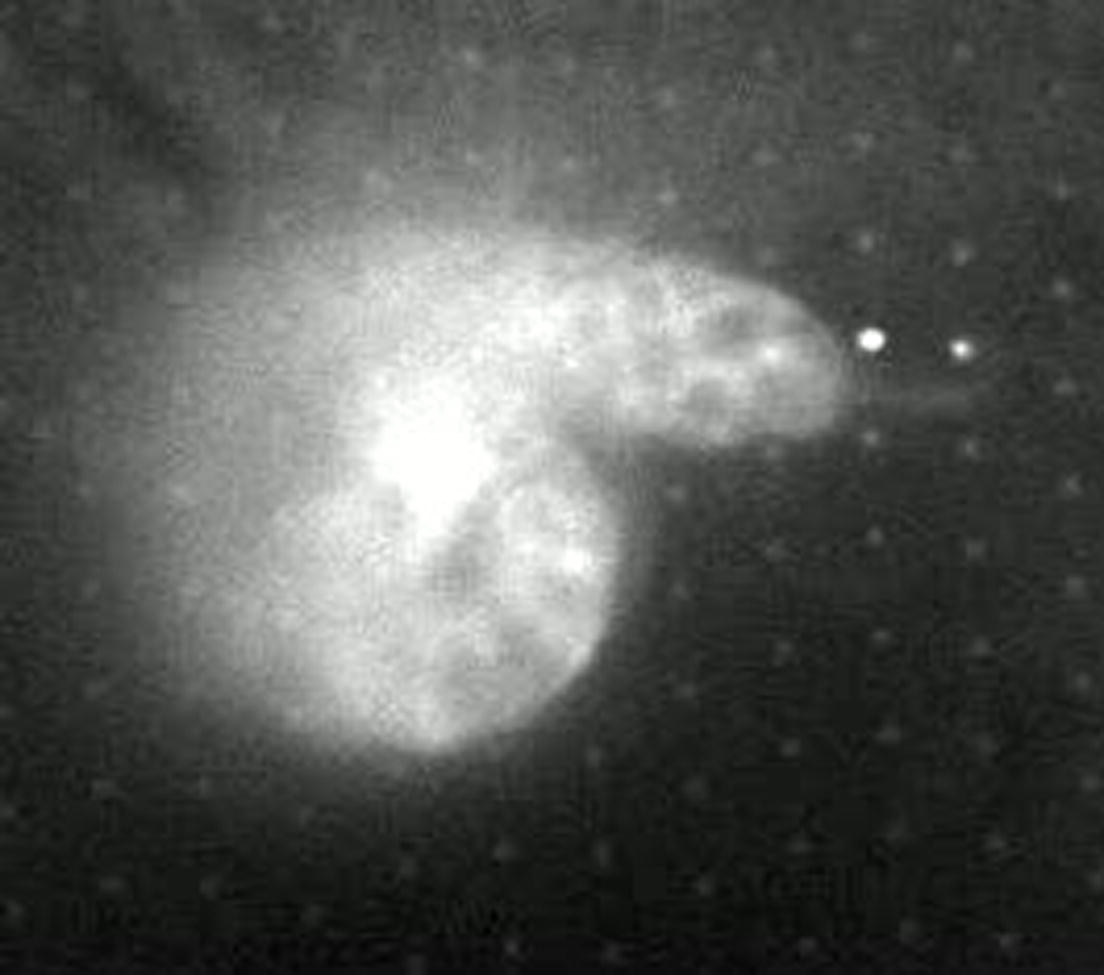

Supplement: Movie S4. Laser Ablation of Anterior Tip Cells, Related to Figure 2 — Laser ablation of a stage 13 anterior tip cell (arrow) expressing membrane CD8-GFP driven by ctB-Gal4. The tubule “recoils” anteriorly immediately after tip cell ablation. [file mmc5.jpg]

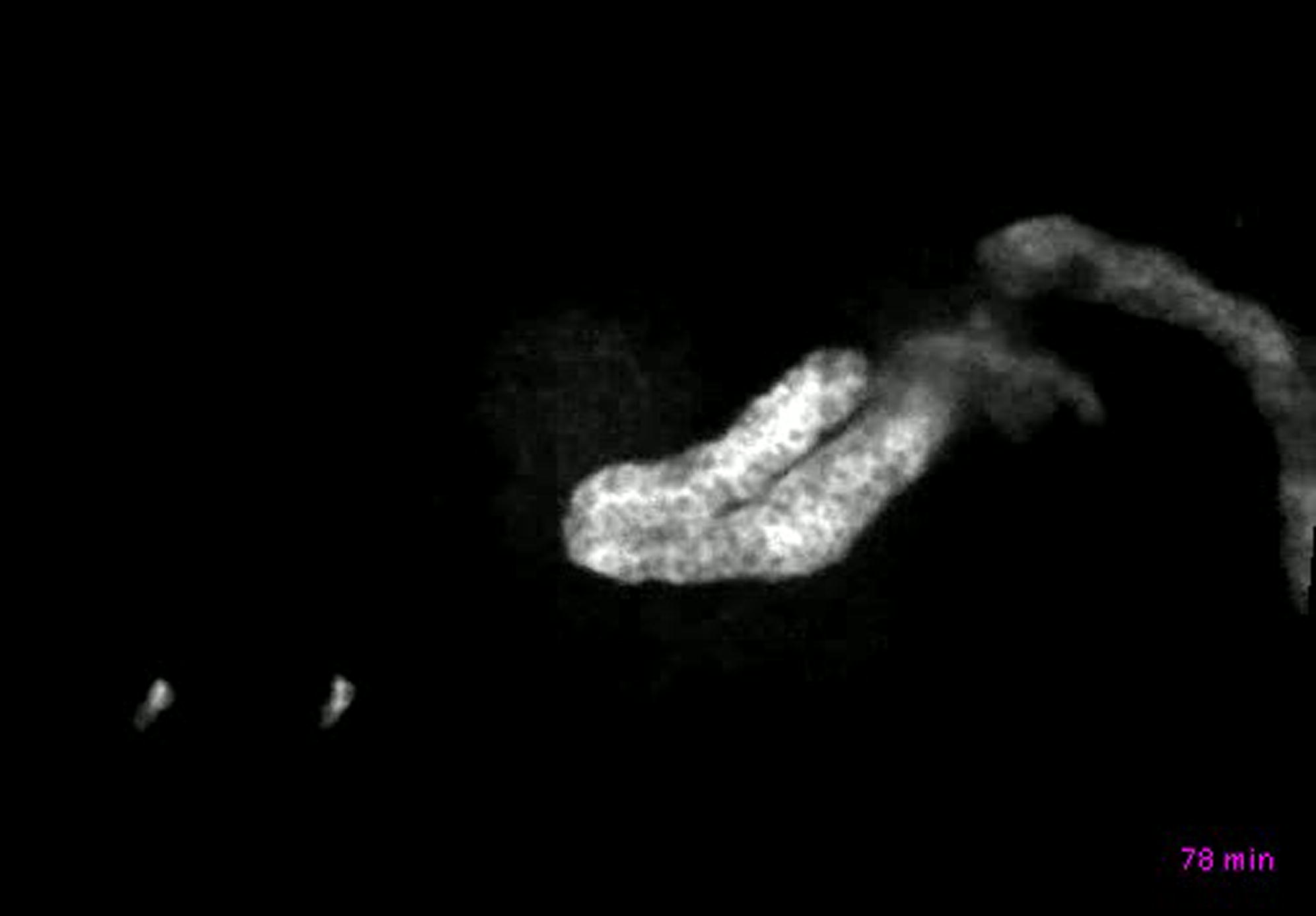

Supplement: Movie S5. Tip Cell Ablation Perturbs Anterior Tubule Shape and Position, Related to Figure 2 — Anterior tubules (ctB-Gal4>UAS-mCD8-GFP) lose their stereotypical shape after tip cell ablation; the distal tubule ends move further anteroventrally and the kink region shifts distally. Images were taken every 3 min. [file mmc6.jpg]

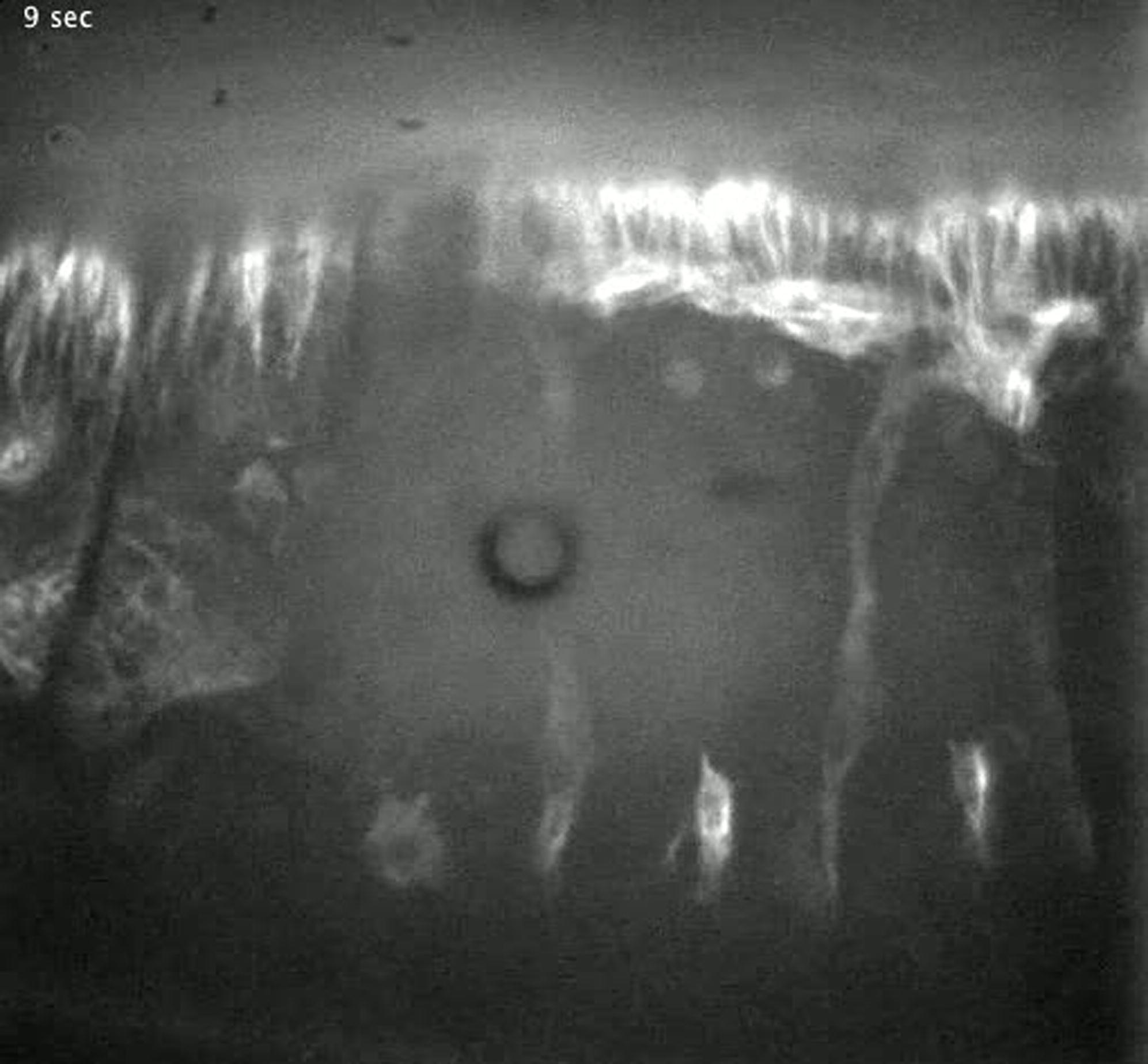

Supplement: Movie S6. Laser Ablation of Embryonic Alary Muscles, Related to Figure 3 — Alary muscle ablation (A4/A5; membrane CD8-GFP driven by tup-Gal4) in a stage 14 embryo. [file mmc7.jpg]
